# Synthesis, Characterization, Biological Activity and Molecular Docking Studies of Novel Organotin(IV) Carboxylates

**DOI:** 10.3389/fphar.2022.864336

**Published:** 2022-04-05

**Authors:** Niaz Muhammad, Mukhtar Ahmad, Muhammad Sirajuddin, Zafar Ali, Nikolay Tumanov, Johan Wouters, Abdelbasset Chafik, Kübra Solak, Ahmet Mavi, Shabbir Muhammad, Shaukat Shujah, Saqib Ali, Abdullah G. Al-Sehemi

**Affiliations:** ^1^ Department of Chemistry, Abdul Wali Khan University, Mardan, Pakistan; ^2^ Department of Chemistry, University of Science and Technology, Bannu, Pakistan; ^3^ NISM, NARILIS, Université de Namur, Namur, Belgium; ^4^ Ecole Supérieure de Technologie, Université Cadi Ayyad, El Kelâa des Sraghna, Morocco; ^5^ Laboratoire Bioressources et Sécurité Sanitaire des Aliments, Faculté des Sciences et Techniques, Université Cadi Ayyad, Marrakech, Morocco; ^6^ Department of Nanoscience and Nanoengineering, Institute of Science, Atatürk University, Erzurum, Turkey; ^7^ Department of Nanoscience and Nanoengineering, Graduate School of Natural and Applied Sciences, Atatürk University, Erzurum, Turkey; ^8^ Department of Mathematics and Science Education, Education Faculty of Kazım Karabekir, Atatürk University, Erzurum, Turkey; ^9^ Department of Chemistry, College of Science, King Khalid University, Abha, Saudi Arabia; ^10^ Department of Chemistry, Kohat University of Science and Technology, Kohat, Pakistan; ^11^ Department of Chemistry, Quaid-I-Azam University, Islamabad, Pakistan

**Keywords:** organotins, single crystal XRD, antibacterial activity, anticancer activity, molecular docking studies

## Abstract

Four new carboxylates complexes with general formula R_2_SnL_2_ and R_3_SnL, where R = *n*-butyl (**1**, **3**), methyl (**2**, **4**) and L = 4-Chlorophenoxyacetate, were synthesized in significant yields. FT-IR analysis revealed a chelating (**1** and **2**) and a bridging bidentate (**3** and **4**) coordination modes for the carboxylate ligand in solid state which was further confirmed by the single crystal X-ray analysis of complex **4**. The NMR data (^1^H, ^13^C and ^119^Sn) revealed a higher coordination number around the tin center in R_2_SnL_2_ (**1** and **2**) compared to R_3_SnL (**3** and **4**). A close matching was observed between the experimental and calculated structures (obtained at B3LYP/6-31G* + LANL2DZ basis set). Quantum chemical analysis indicates that the carboxylate moiety has the major contribution in the formation of filled and unfilled orbitals as well as in ligand to ligand intramolecular charge transfer during the electronic transitions. The cytotoxicity data of the screened compounds evaluated against lung cancer cell line (A549) and normal lung fibroblast cell line (MRC-5) revealed that **1**, **3** and **4** have shown dose dependent cytotoxic effects while **HL** and **2** have shown steady and low cytotoxic activities. The antibacterial activity of complexes **1–4** is higher than that of **HL**. Molecular docking study showed an intercalation binding mode for complex **3** with DNA (docking score = −3.6005) involving four polar interactions. Complex **3** docking with tubulin (PDB ID 1SA0) with colchicine as a target protein resulted in three polar interactions (docking score −5.2957). Further, the docking analysis of the **HL** and **1–4** has shown an adequate interactions with the coronavirus SARS-CoV-2 spike protein, nucleocapsid protein and human angiotensin converting enzyme (ACE2).

## 1 Introduction

Cancer is the most threating disease of the present time. In spite of extensive research in the field, only few drugs are available commercially. *Cis*-platin being the lead one is associated with some severe side effects like neuro-, nephro-, and ototoxicity (Mubeen and Kini, 2012; Pantelić et al., 2019) which motivates the researchers for developing of non-platinum based anticancer drugs. Organotin(IV) carboxylates are found be the most promising alternatives, as these have shown higher apoptosis inducing character compared to the platinum complexes, both *in vivo* and *in vitro* (Indumathy et al., 2007; Butt et al., 2019). In addition to the anticancer potential, organotin(IV) carboxylates also possess significant antibacterial, antifungal, insecticidal, anti-tuberculosis and antidiabetic activities (Kovala-Demertzi et al., 2009; Tzimopoulos et al., 2010; Tariq et al., 2013; Sirajuddin et al., 2014a; Sirajuddin et al., 2014b; Shah et al., 2015; Tariq et al., 2016; Debnath et al., 2019; Sirajuddin et al., 2021).

In spite of wide range available data on the anticancer activity of organotin(IV) carboxylates, exact conclusive results are not available on the cytotoxic effects of these complexes. Some possible targets identified for the anticancer activity of the organotin(IV) complexes are discussed in the form nucleotide base pairs, nitrogen atoms of DNA, phosphate groups on the backbone of DNA and the sulfhydryl groups on proteins (Gielen, 2008; Hong et al., 2015; Xiao et al., 2020).

For an effective interaction with these possible target sites and other bioactivities, the coordination expansion ability of Sn atom, number and nature of R groups bonded to Sn and nature of the carboxylate ligand are found the important controlling factors (Saxena and Huber, 1989; Hook, 1994; Gielen, 2003; Hadjikakou and Hadjiliadis, 2009; Sirajuddin et al., 2012). The nature of carboxylate ligand is quite important in deciding the bioactivity of the organotin(IV) complexes as it can alter hydro/lipophilicity of the complex and assist the transportation of complexes across the cell membrane (Cooney and Wuertz, 1989; Yenişehirli et al., 2010; Ettouhami et al., 2012). Also for the same ligand the earlier reported toxicity order is as: R_3_Sn > R_2_Sn > RSn. (Basu, 2008). In addition, the biological target (microorganism and stain, nature of the cancer cell lines) also significantly affect the activity as well.

A. Lavecchia, et al., investigated the screening results for a series of chiral phenoxyacetic acid analogues agonist activity toward the human PPARα (hPPARα) and PPARγ (hPPARγ) subtypes and found some of analogues are potent PPARα agonists as well as PPARγ agonists. Interestingly, for one of these analogues, the stereoselectivity toward PPARα was reversed which was further confirmed by docking experiments to rationalize this peculiar behavior (Fracchiolla et al., 2007). Santiago Gómez-Ruiz and Goran N. Kaluđerović et al., reported the cytotoxic activity of phenoxyacetic acid based di and triphenyltin(IV) derivatives against tumor cell lines human adenocarcinoma HeLa, human myelogenous leukemia K562, human malignant melanoma Fem-x and normal immunocompetent cells, peripheral blood mononuclear cells PBMC. It is evidenced form their study that triphenyltin(IV) complexes show higher activities than the diphenyltin(IV) derivatives and some of the invested compounds are more potent than *cis*-platin against all the tested cells and relative high selectivity especially on K562 cells (Gómez-Ruiz et al., 2008; Kaluđerović et al., 2010). Tariq Ali et al., studied *via* docking studies the interactions of 2-chlorophenyl ethanoic acid based organotin(IV) complexes against the SARS-Co-2 virus which is responsible for the most challenging disease Covid-19 and found a considerable binding interactions of compounds with the angiotensin-converting enzyme 2 (ACE2) and with the nucleocapsid protein of the SARS- CoV-2 virus.

Thus keeping in view the biological importance of the phenoxyacetic acid derivatives especially against SARS- CoV-2 virus, here we have chosen the 4-chlorophenoxyacetate for the preparation of organotin(IV) complexes. The aim of the present study is to described the synthesis, characterization, *in vitro* anticancer and antibacterial activity of di- and tri-*n*-butyl/methyltin(IV) derivatives of 4-chlorophenoxy acetic acid. The interaction of the synthesized compounds with DNA, spike protein, nucleocapsid protein of coronavirus SARS-CoV-2 and human angiotensin converting enzyme (ACE2) was also studied using molecular docking.

## 2 Experimental

### 2.1 Materials and Physical Measurements

Trimethyltin chloride (1,498+%), tri-*n*-btyltin chlorides (96%), dimethyltin dichlorides (98%), di-*n*-butyltin dichlorides (96%), 4-chlorophenoxyacetic acid (98+%), sodium bicarbonate (99.7%) and analytical grade chloroform (99.8+%) were purchased from commercial suppliers and used as such without any further purification. However, chloroform was dried by a reported method (Armarego and Chai, 2003).

Lung cancer cell line (A549) and Normal lung fibroblast cell line (MRC-5 An1) were provided by Eastern Anatolia High Technology Application and Research Center (DAYTAM, Erzurum, Turkey) and Ministry of Agriculture and Forestry (şap institute, Turkey), respectively. 3-(4,5-dimethylthiazol-2-yl)-2.5-diphenyl tetrazolium bromide (MTT, M2128) was purchased from Sigma-Aldrich. Roswell Park Memorial Institute (RPMI) medium, Trypsin/Ethylenediaminetetra acetic acid (EDTA) solution, fetal bovine serum (FBS), L-glutamine were purchased from Gibco. Dimethylsulfoxide (DMSO) (cell culture reagent) was purchased from Santa Cruz Biotechnology.

A Gallenkamp (United Kingdom) electro thermal instrument was used to find the melting points of the synthesized compounds. Elemental analysis was done on Leco CHNS 932. FT-IR spectra (4,000–400 cm^−1^) were recorded on a Nicolet-6700 FT-IR spectrophotometer, Thermoscientific, United States. The NMR (^1^H, ^13^C and ^119^Sn) spectra were obtained at room temperature on a Bruker Advance Digital 300 MHz NMR spectrometer (Switzerland). Single-crystal X-ray diffraction data was collected using the Oxford Diffraction Gemini R Ultra diffractometer (Mo Kα, graphite monochromator, Ruby CCD area detector) at 295 (2) K.

### 2.2 Synthesis

**SCHEME 1:**
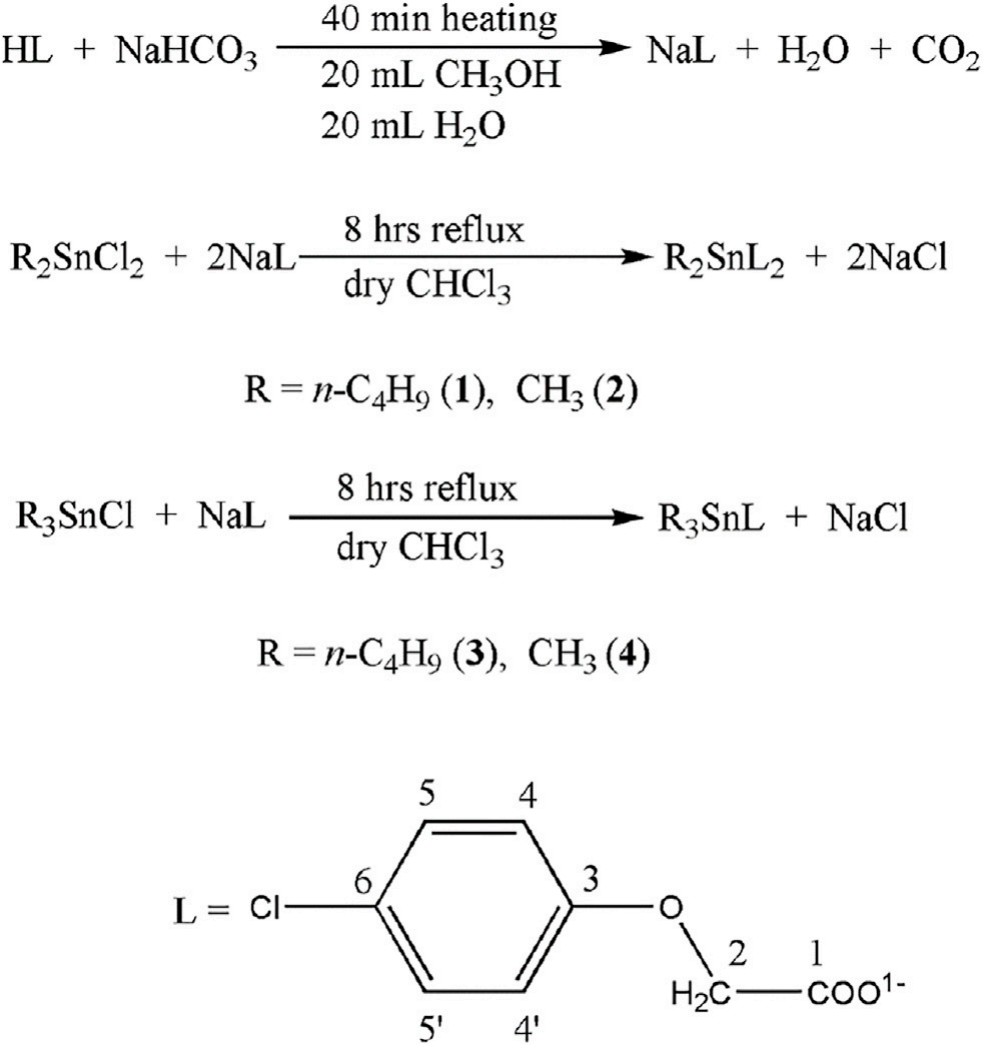
Synthetic procedure for NaL and complexes **1**–**4**.

Synthesis of the ligand salt (**NaL**) and complexes (**1–4**) is shown in Scheme 1 and is discussed as follow.

#### 2.2.1 Sodium-4-Chlorophenoxyacetate (NaL)

The ligand salt (NaL) was prepared by adding dropwise 20 ml aqueous sodium bicarbonate solution (20 mmol) to the 20 ml methanolic solution of 4-chlorophenoxyacetic acid, **HL** (20 mmol). The mixture was heated for 40 min at 60°C. The soluble salt (**NaL**) was recovered from the solution as a white solid by rotary evaporation and dried under vacuum.

#### 2.2.2 Di-*n*-Butyltin(IV)-*Bis*-(4-Chlorophenoxy Acetate) (1)

For the synthesis of complex **1**, the ligand salt (1.043 g, 5 mmol) and (*n*-C_4_H_9_)_2_SnCl_2_ (0.760 g, 2.5 mmol) were dissolved in 100 ml dry chloroform contained in a round bottom flask. The mixture was refluxed for 8 h. The resultant turbid mixture was cooled to a room temperature and left undisturbed for 24 h. The precipitated sodium chloride was removed from the mixture by filtration. The filtrate was evaporated under reduced pressure by rotary evaporator. The remaining solid crude product was recrystallized from chloroform and *n*-hexane mixture (4:1).

Chemical formula: C_24_H_30_Cl_2_O_6_Sn, Molecular Mass: 604.04, Percent yield: 84%. m.p.: 85–87°C. Anal Calc. (experimental values) %: C = 47.72 (47.69); H = 5.01 (5.03). FT-IR (cm^−1^): 1602s υ(COO_asym_), 1488s υ(COO_sym_), 114 Δυ, 504 m υ(Sn-C), 481w υ(Sn-O). ^1^H-NMR (CDCl_3_, ppm): 4.68 (s, 4H, H-2), 7.25 (d, 4H, H-4,4′), 6.85 (d, 4H, H-5,5′), 1.78–1.63 (m, 4H, H-α), 1.78–1.63 (m, 4H, H-β), 1.44–1.28 (m, 4H, H-γ), 0.94 (t, 6H, H-δ). ^13^C-NMR (CDCl_3_, pm): 177.7, 65.34, 156.2, 115.8, 129.5, 126.7 (C-1 to C-6, respectively), 28.2, 26.6, 26.4, 13.6 (C-α, β, γ, δ, respectively). ^119^Sn-NMR (CDCl_3_, ppm): −228.

#### 2.2.3 Dimethyltin(IV)-*Bis*-(4-Chlorophenoxy Acetate) (2)

Complex **2** was synthesized and recrystallized by the same procedure as used for complex **1**, just by changing (CH_3_)_2_SnCl_2_ (0.549 g, 2.5 mmol).

Chemical formula: C_18_H_18_Cl_2_O_6_Sn, Molecular Mass: 519.95, Percent yield: 80%. m.p.: 180–181°C. Anal Calc. (experimental values) %: C = 41.58 (41.56); H = 3.49 (3.46). FT-IR (cm^−1^): 1609s υ(COO_asym_), 1488s υ(COO_sym_), 121 Δυ, 583s υ(Sn-C), 445 m υ(Sn-O). ^1^H-NMR (CDCl_3_, ppm), [^2^ *J* (^119/117^Sn, ^1^H) in Hz]: 4.60 (s, 4H, H-2), 7.29 (d, 4H, H-4,4′), 6.91(d, 4H, H-5,5'), 0.84 (s, 6H, H-α) (101). ^13^C-NMR (CDCl_3_, pm): 172.1, 65.7, 157.2, 116.6, 129.5, 124.9 (C-1 to C-6, respectively), 4.1 (C-α). ^119^Sn-NMR (CDCl_3_, ppm): −220.

#### 2.2.4 Tri-*n*-Butyltin(IV)-4-Chlorophenoxy Acetate (3)

Complex **3** was made by the same procedure as used for complex **1**, except equimolar amounts of the reactants are taken, i.e., NaL (0.522 g, 2.5 mmol), (*n*-C_4_H_9_)_3_SnCl (0.814 g, 2.5 mmol).

Chemical formula: C_20_H_33_ClO_3_Sn, Molecular Mass: 476.11, Percent yield: 82%. m.p.: 50–51°C. Anal Calc. (experimental values)%: C = 50.50 (50.47); H = 6.99 (6.88). FT-IR (cm^−1^): 1580s υ(COO_asym_), 1409s υ(COO_sym_), 171 Δυ, 566 m υ(Sn-C), 499 m υ(Sn-O). ^1^H-NMR (CDCl_3_, ppm): 4.54 (s, 2H, 2), 7.18 (d, 2H, 4,4′), 6.79 (d, 2H, 5,5′), 1.59 (t, 6H, Hα), 1.37–1.24 (m, 6H, Hβ), 1.37–1.24 (m, 6H, Hγ), 0.88 (t, 9H, Hδ). ^13^C-NMR (CDCl_3_, pm), [^n^ *J* (^119^Sn, ^13^C) in Hz]: 173.3, 65.8, 156.7, 115.8, 129.2, 126.0 (C-1 to C-6, respectively), 16.9 (343), 27.7 (21), 26.9 (64), 13.6 (C-α, β, γ, δ, respectively). ^119^Sn-NMR (CDCl_3_, ppm): −50.

#### 2.2.5 Trimethyltin(IV)-4-Chlorophenoxy Acetate (4)

Complex **4** was made by the same procedure as used for complex **3**, just by changing (CH_3_)_3_SnCl (0.498 g, 2.5 mmol).

Chemical formula: C_11_H_15_ClO_3_Sn, Molecular Mass: 349.97, Percent yield: 88%. m.p.: 160–162°C. Anal Calc. (experimental values) %: C = 37.81 (37.79); H = 4.33 (4.29). FT-IR (cm^−1^): 1590s υ(COO_asym_), 1432s υ(COO_sym_), 158 Δυ, 552s υ(Sn-C), 452 m υ(Sn-O). ^1^H-NMR (CDCl_3_, ppm), ^2^ *J* [(^119^Sn, ^1^H), Hz]: 4.55 (s, 2H, H-2), 7.24 (d, 2H, H-4,4′), 6.84 (d, 2H, H-5,5′), 0.61 (s, 9H, H-α) (59). ^13^C-NMR (CDCl_3_, pm), ^1^ *J* [(^119^Sn, ^13^C) in Hz]: 173.4, 65.8, 156.6, 115.9, 129.4, 126.2 (C-1 to C-6, respectively), −1.9 (C-α) (394). ^119^Sn-NMR (CDCl_3_, ppm): −30.

### 2.6 Single Crystal X-Ray Diffraction Analysis

Data collection, unit cells determination and data reduction were carried out using CrysAlis PRO software package (Rigaku, 2018). Using Olex2 and shelXle, the structure was solved with the SHELXT 2015 structure solution program by Intrinsic Phasing methods and refined by full-matrix least squares on |F|^2^ using SHELXL-2018/3 (Sheldrick G., 1997; Sheldrick, 1997b; Dolomanov et al., 2009; Sheldrick, 2009; Hübschle, 2011; Scheldrick, 2015). Non-hydrogen atoms were refined anisotropically. Hydrogen atoms were placed on calculated positions in riding mode with temperature factors fixed at 1.2 times *U*_eq_ of the parent carbon atoms (1.5 times for methyl groups). CCDC deposition number for **4** is 2128461.

### 2.7 Biological Activity

#### 2.7.1 *In Vitro* Cytotoxicity of Ligand Acid HL and Complexes 1–4

The cytotoxicity test of the **HL** and complexes **1–4** was performed against lung cancer cell line (A549) and normal lung fibroblast cell line (MRC-5). Cells were counted by trypan blue staining and seeded in 96 well-plate with density of 5–10 × 10^3^ cells/well in 0.15 ml of RPMI media containing 10% fetal bovine serum and 1% L-glutamine. The cells were then incubated for 24 h at 37°C (5% CO_2_, 90% humidity). Cell viability was analyzed using the MTT assay after treatment for 24 and 48 h with different concentrations (0, 0.1, 0.5, 1, 2, 5, 10 and 50 μg/ml) of the screened compounds. After the removal of the culture medium the cells were subsequently treated with 10 μL of 5 mg/ml thiazolyl blue tetrazolium bromide in 90 μL of media (Pettazzoni et al., 2011; Abu-Dief et al., 2021a; Saddik et al., 2021). After 2 h, 100 μL of DMSO was used to dissolve MTT salt. The absorbance was recorded at 570 nm by a plate reader (Biotek). Values from three wells of cells from the same preparation were averaged as a single value for that experiment. Analysis of variance (ANOVA) test was used to determine the difference between the groups, *p* < 0.05 was considered statistically significant.

#### 2.7.2 Antibacterial Activity

The antibacterial activity of the screened compounds was evaluated through a reported procedure (Bagamboula et al., 2003; Abu-Dief et al., 2019; Abu-Dief et al., 2020; Abu-Dief et al., 2021b). At 121°C for 30 min, micro tips, media flask, test tube, swabs, Petri plates and all other equipment were sterilized in an autoclave. 25 ml of the nutrient media was poured into each Petri plate and was then allowed to cool. Three bacterial strains (*E. coli, K. pneumonia* and *S. aureus*) were streaked on the nutrient agar plates with sterile swabs. Five wells were punched in each Petri plate with sterile borer of 6 mm distance across. Stock solutions (3 mM) of the test compounds were prepared in DMSO which were further diluted to 100, 200 and 300 μg/ml concentrations. 20 µL of each solution was added to the particular well. DMSO and Ciprofloxacin (10 μg/ml) serving as negative and positive controls were also loaded. All the plates were placed in incubator at 37°C for 24 h and zones of inhibitions were measured in mm.

### 2.8 Computational Methods

#### 2.8.1 Quantum Chemical Analysis

All the quantum chemical calculations were executed through the use of Gaussian 16 suit of programs (Frisch et al., 2016). The B3LYP functional is used among the available DFT methods (Stephenspj and Chabalowskic, 1994). A mixed basis set (6-31G* + LANL2DZ) consisting of LANL2DZ for Sn metal atom and 6-31G* for all remaining atoms was used for all calculations (Chiodo et al., 2006). Such type of mixed basis set are found reasonable for metal complexes in several previous reports (Yang et al., 2009). The total density and partial density of states were determined using a full scale population method at the same level of theory. GaussView 6 was used for getting the 3D orbital diagrams.

#### 2.8.2 Molecular Docking Studies

The 3D molecular structures of the ligand acid HL and synthesized complexes 1–4 were drawn and optimized using Molecular Operating Environment (MOE-2016) software (Inc, 2016). From the protein data bank (www.rcsb.org/pdb) the x-ray crystallographic structures of the double stranded DNA (PDB ID: 1BNA) and tubulin ((PDB ID: 1SA0) in complex with colchicine as protein structure were retrieved as possible targets for the anticancer activity of the synthesized complexes. Also, the crystal structures of angiotensin converting enzyme of human body, nucleocapsid protein and spike protein of SARS-CoV-2 were retrieved from the same source. From the crystal structures all the water molecules were removed using MOE-2016 software (www.chemcomp.com). The 3D protonation and energy minimization were done using default parameters of Molecular Operating Environment. All the test compounds were docked with PDB files using default parameters of MOE-2016 (Method- Alpha PMI, Rigid receptor, score- Affinity dG, and poses-10).

## 3 Results and Discussions

Organotin(IV) derivatives (**1–4**) were made by reacting organotin(IV) chlorides with sodium-4-chlorophenoxyacetate (NaL) at a mole ratio that was appropriate. In dry chloroform, the reaction mixtures were refluxed for 8 h. As crystalline/powdered products, the complexes were produced in a high yield (>80%). At ambient temperature, the complexes are soluble in DMSO and CHCl_3_.

### 3.1 FT-IR Spectroscopy

The FT-IR spectra of the complexes **1–4** was compared with that of the free ligand acid (HL) for the preliminary structural identification. The lack of a wide band in the higher wave number region above 2,400 cm^−1^, attributable to the hydroxyl group of the COOH moiety in the free carboxylic acid, was a notable difference in the spectra of the complexes (Muhammad et al., 2009). The absence of this peak indicated the presence of deprotonated coordinated carboxylate moiety in the complexes (Muhammad et al., 2009). The presence of several additional weak/medium intensity bands in the spectra of the complexes below 500 cm^−1^ owing to Sn-O bond formation bolstered the claim.^27^ The existence of a set of two peaks in the ranges 1,609-1,580 cm^−1^ and 1,488-1,409 cm^−1^ for complexes **1–4**, corresponding to asymmetric and symmetric COO vibrations of the coordinated ligand, respectively, was another notable aspect of the spectra of the complexes (Muhammad et al., 2019). In complexes **1-2** and **3-4**, the difference (Δυ) of these two bands suggested a chelating and a bridging bidentate coordination mode of the carboxylate moiety, respectively (Muhammad et al., 2009). Single-crystal X-ray diffraction study backed up the structural assumptions provided by FT-IR spectra for the complex **4**. The representative FT-IR spectra of complexes **1** and **4** are given in Supplementary Figures 1, 2, respectively.

### 3.2 NMR Spectroscopy

^1^H, ^13^C and ^119^Sn NMR spectra obtained in CDCl_3_ and DMSO were used to analyze the solution state structural analyses of the complexes **1–4**. The ^1^H NMR spectra showed no singlet signal above 10 ppm, indicating the existence of a deprotonated carboxylate ligand in the complexes (Muhammad et al., 2019). A singlet was observed for the -CH_2_- protons (at position 2) in the range 4.68–4.54 ppm. An important observation for this signal is that, in case of diorganotin (IV) derivatives (**1** and **2**) the signal appeared in the down field region (at 4.68 ppm for **1** and at 4.60 ppm for **2**) compared to the triorganotin (IV) derivatives (at 4.55 ppm for **3** and at 4.54 ppm for **4**). This observation clearly indicates the coordination mode differences between di- and triorganotin(IV) derivatives. In case of diorganotin(IV) complexes (**1** and **2**) a bidentate mode of coordination can be observed in solution (Muhammad et al., 2019). This makes the carbonyl carbon (Cooney and Wuertz) and subsequently the methylene group (-CH_2_-) more de-shielded. However, in case of triorganotin(IV) derivatives a monodentate behavior of the carboxylate ligand causes less de-shielding of the –OOC–CH2– fragment, so methylene signal appears a bit up field. The benzene ring protons of the ligand appeared as two doublets in the ranges 7.29–7.18 ppm and 6.91–6.79 ppm for 4,4′ and 5,5′, protons respectively. Complexes **2** and **4** gave clear singlets for tin bonded methyl groups at 0.84 and 0.61 ppm, respectively. The ^2^ *J* (^119^Sn,^1^H) coupling constant values for these signals were found to be 101 and 59 Hz for **2** and **4** respectively. These values confirm hexa and tetra-coordinated tin cneter in solution for **2** and **4**, respectively (Muhammad et al., 2019). In complexes **1** and **3**, the *n*-butyl groups have shown a complicated pattern in form of multiplets for the –CH2–CH2–CH2 skeleton. However, clear triplets were observed for the terminal CH_3_ groups in both the complexes.

The ^13^C-NMR spectra of complexes revealed six signals in the predicted locations for the magnetically non-equivalent carbons of the carboxylate ligand. By comparing the signals of the produced complexes to those of comparable reported tin carboxylate complexes, the carbons of the tin bound alkyl groups were allocated (Muhammad et al., 2014). Four coordinated tetrahedral tin centres have been verified in complexes **3** and **4** using ^1^ *J* (^119^Sn,^13^C) coupling constant values of 343 and 394 Hz, respectively (Muhammad et al., 2014). In the carbon NMR spectra of all produced compounds, no additional peak was identified. This represented the pure character of synthesised complexes.

The ^119^Sn NMR of the reported compounds gave a sharp single (Supplementary Figure 2) and fall in the range of hexa-coordinated (for complexes **1** and **3**) and tetra-coordinated (for complexes **2** and **4**) environment around the Sn atom (Sirajuddin et al., 2014a).

The representative ^1^H, ^13^C and ^119^Sn NMR spectra of complexes **2**, **3** and **4** are given in Supplementary Figures 3–9.

### 3.3 Description of Complex 4 Crystal Structure

**FIGURE 1:**
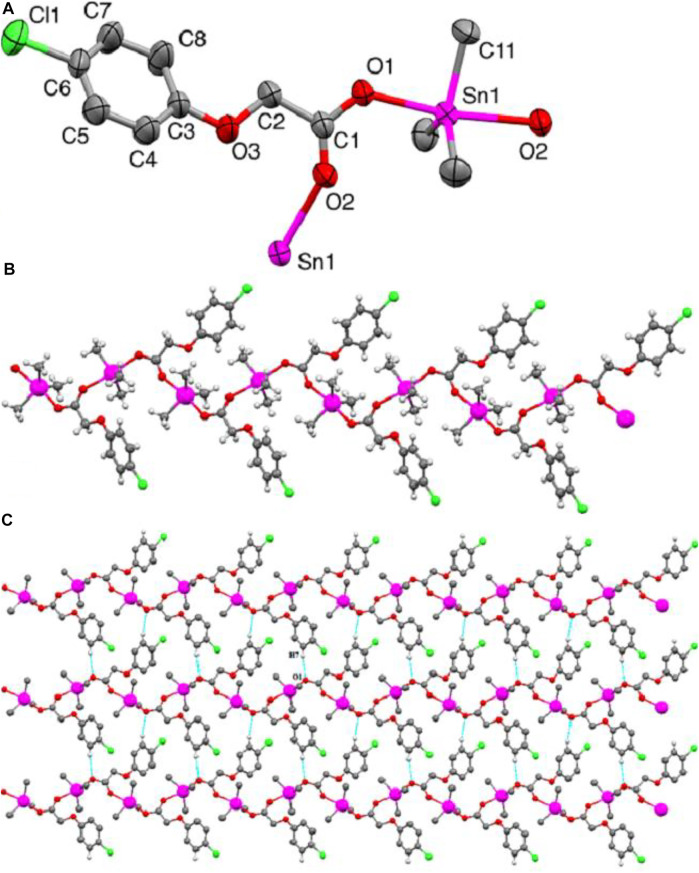
**(A)** ORTEP view of 4 with a 50% probability level **(B)** 1-D zigzag polymeric chain structure. **(C)** 2-D polymeric network. H-atoms non-involved in hydrogen bonding are omitted for clarity.

The molecular structure obtained from the single-crystal X-ray diffraction analysis of complex **4** is shown in Figure 1A. The complex crystallizes in the monoclinic crystal system with a *P*2_1_/*c* space group. The important crystallographic parameters, selected bond lengths and bond angles are presented in Tables 1, 2, respectively. Complex **4** adopted an infinite zig-zag 1-D polymeric chain structure consisting mono deprotonated bridging carboxylate ligands connecting the tin atoms as shown in Figure 1B. The Sn1-O1 and Sn1-O2 bond distances are 2.2018 (16) Å and 2.3803 (17) Å, respectively showing a slight asymmetric coordination of the carboxylate ligand to the tin atom. The shorter tin-oxygen bond is associated with a longer C-O bond and vice versa. The Sn–O bond distances are more close to the sum of the covalent radii of tin and oxygen (2.13 Å), but are considerably less than the van der Waals radii (3.69 Å), indicating a strong Sn–O bonding interactions (Barsan et al., 2008; Shujah et al., 2014; An et al., 2020). The Sn-O bonded distances are comparable to the other trimethyltin (IV) carboxylate derivatives previously reported (Iqbal et al., 2013). Sn1-C9, Sn1-C10 and Sn1-C11 bond distances are 2.110 (3)Å, 2.108 (3)Å and 2.123 (3)Å, respectively. The tin atom in complex **4** has a five coordinated trigonal bipyramidal geometry. The two axial sites of the trigonal bipyramid are occupied by the oxygen atoms of the bridging carboxylate ligands with an O2-Sn1-O1 bond angle of 171.49 (6)°, showing distorted geometry around tin center. The three tin bonded methyl groups form the equatorial plane of the trigonal bipyramid. The sum of equatorial CH_3_-Sn-CH_3_ bond angles is 359.8° which indicates that the three methyl groups are nearly coplanar and more symmetrically bonded compared to the oxygen atoms of the carboxylate ligands. The three coplanar methyl groups are slightly tilted towards the longer tin-oxygen bond (Sn1-O2) shown by the smaller C-Sn-O2 angle (mean bond angle = 88.53°) compared to the larger C-Sn-O1 angle (mean bond angle = 91.34°). The distortion in pentagonal bipyramidal geometry was also quantitatively calculated by Addison tau-parameter (τ = β–α/60, where β is the largest and α is the second largest angle around tin atom) (Addison et al., 1984). The τ value for a perfect trigonal-bipyramidal and square- pyramidal geometry is 1 and 0, respectively for five-coordinated metal centers. The calculated τ value for the complex **4** is 0.81. This indicates a distorted trigonal bipyramidal geometry around Sn center. Furthermore, the coordination polymers of the complex **4** are connected via relatively weaker C7-H7-----O1 (2.592) interactions resulting in a 2-D polymeric network as shown in Figure 1C.

**TABLE 1:** Important crystallographic parameters of **4**.

Empirical formula	C_11_H_15_O_3_SnCl
F. weight	349.37
Temp./K	295 (2)
Crystal system	Monoclinic
Space group	*P*2_1_/*c*
a/Å	10.7644 (3)
b/Å	10.5041 (2)
c/Å	12.3762 (4)
α, γ/°	90
β/°	95.623 (3)
V/Å^3^	1,392.64 (7)
Z	4
ρ_calc_g/cm^3^	1.666
μ/mm^−1^	2.017
F (000)	688.0
Crystal size/mm^3^	0.67 × 0.41 × 0.29
Radiation	Mo Kα (λ = 0.71073)
2θ range for data collection/°	5.096 to 61.016
Index ranges	−10 ≤ h ≤ 15, −14 ≤ k ≤ 15, −17 ≤ l ≤ 14
Ref. collected	8,239
Independent ref.	4,248 (R_int_ = 0.0216, R_sigma_ = 0.0412)
Data/restraints/parameters	4,248/0/148
*S*	1.014
Final R indexes [I ≥ 2σ (I)]	R_1_ = 0.0331, wR_2_ = 0.0580
Final R indexes (all data)	R_1_ = 0.0524, wR_2_ = 0.0644
Largest diff. peak/hole/e·Å^−3^	0.33/−0.42

**TABLE 2:** Selected bonds lengths (Å) and bonds angles (°) of complex **4**.

Bonds lengths (Å)			
Sn1-C9	2.111 (3)	C1-O2	1.233 (4)
Sn1-C10	2.107 (3)	C1-C2	1.509 (4)
Sn1-C11	2.123 (3)	C2-O3	1.408 (3)
Sn1-O1	2.202 (2)	C3-O3	1.373 (3)
Sn1-O2	2.380 (2)	C1-O1	1.270 (3)
**Bond angles (°)**			
O2-Sn1-O1	171.49 (6)	C10-Sn1-O1	91.24 (9)
C9-Sn1-C10	122.9 (1)	O1-Sn1-C11	86.98 (9)
C9-Sn1-C11	117.7 (1)	C9-Sn1-O2	88.9 (1)
C10-Sn1-C11	119.2 (1)	C11-Sn1-O2	84.56 (9)
O1-Sn1-C9	95.8 (1)	C10-Sn1-O2	92.14 (9)
O1-C1-O2	125.1 (2)	O1-C1-C2	111.8 (2)

### 3.4 Quantum Chemical Analysis of Complex 4

#### 3.4.1 Optimized Geometry of Complex 4

**FIGURE 2:**
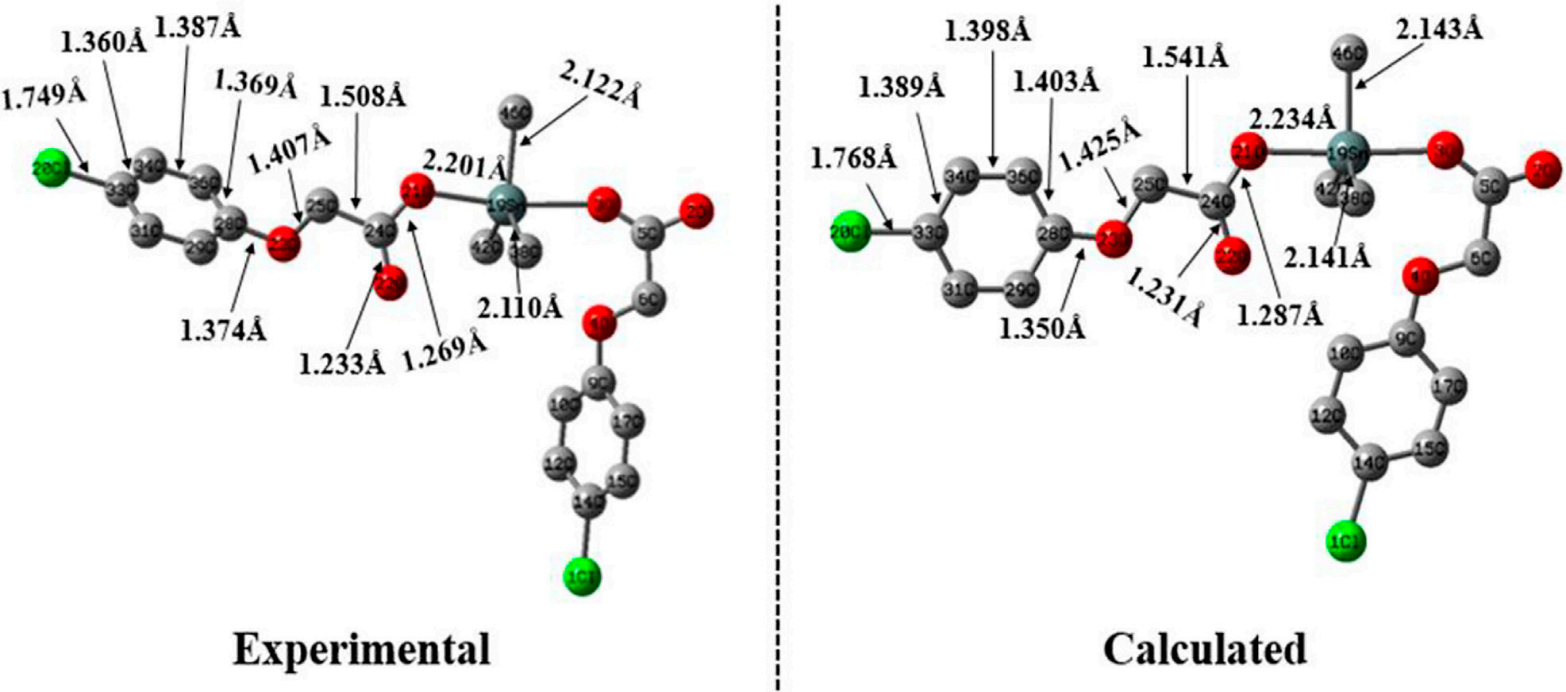
The experimental and calculated geometry of complex **4** computed B3LYP/6-31G* + LANL2DZ basis set.

The optimized geometry of complex **4** is shown in Figure 2 at B3LYP/6-31G* + LANL2DZ basis set. For comparative analysis, the single crystal structure of complex **4** is also presented along with its calculated geometry. An overview of both calculated and experimental structures indicates that there is a reasonably good agreement between them. For more detailed structural comparison, the bonds lengths of only one symmetric unit of left-hand side are compared. For instance, the most important Sn-O_21_ bond lengths are found to be 2.201 and 2.234 Å for experimental and calculated geometries, respectively. Similarly, other important bond lengths of Cl_20_-C_33_ are found to be 1.749 and 1.768 Å for experimental and calculated geometries, respectively.

#### 3.4.2 Total and Partial Density of States

**FIGURE 3:**
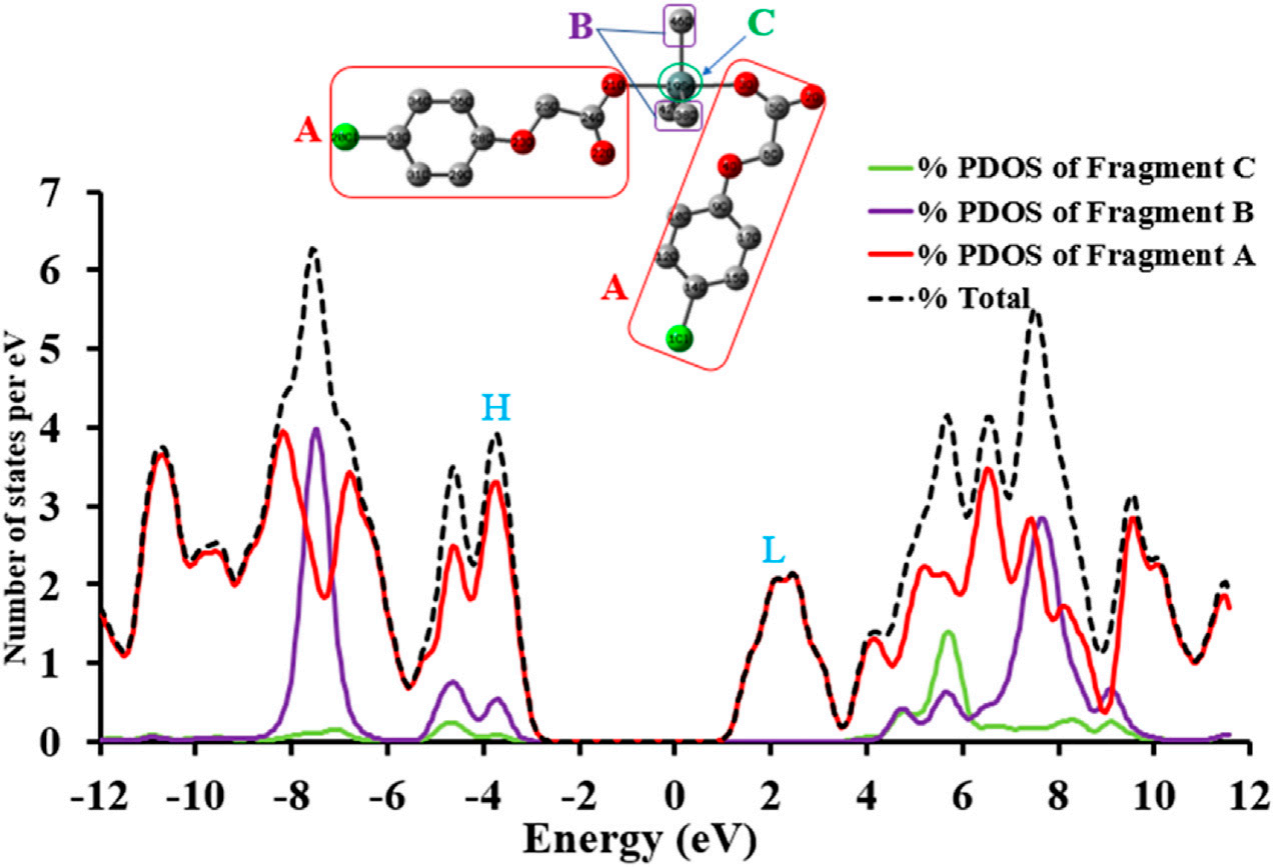
The TDOS and PDOS of complex **4** at B3LYP/6-31G* + LANL2DZ level of theory, where H and L illustrate the energy regions of HOMO and LUMO, respectively.

In order to understand the intrinsic contributions of different moieties of complex to the total density of states, the partial density of states projected over individual moieties of complex **4** are calculated. To obtain the TDOS and PDOS diagrams, the molecules are partitioned into three fragments i.e., fragment A [methyl-2-(4-chlorophenoxy) acetate], fragment B (three methyl groups) and fragment C (Sn metal). It can be observed that Fragment A shows a major percentage contribution both in the formation of filled and unfilled orbitals. According to Figure 3, the percentage contribution of fragment B is maximum in the whole range of energy levels while Fragment B shows maximum percentage contribution in the range of about ∼6.3 to ∼8 eV. The region from −3 to −5 eV is interesting where all fragments relatively contribute including Sn metal atom too. The Sn metal contribution in formation of unfilled orbitals is more (from ∼5 to ∼6 eV) as compared to the states in filled orbitals as can be seen in Figure 3.

#### 3.4.3 Frontier Molecular Orbitals of Complex 4

**FIGURE 4:**
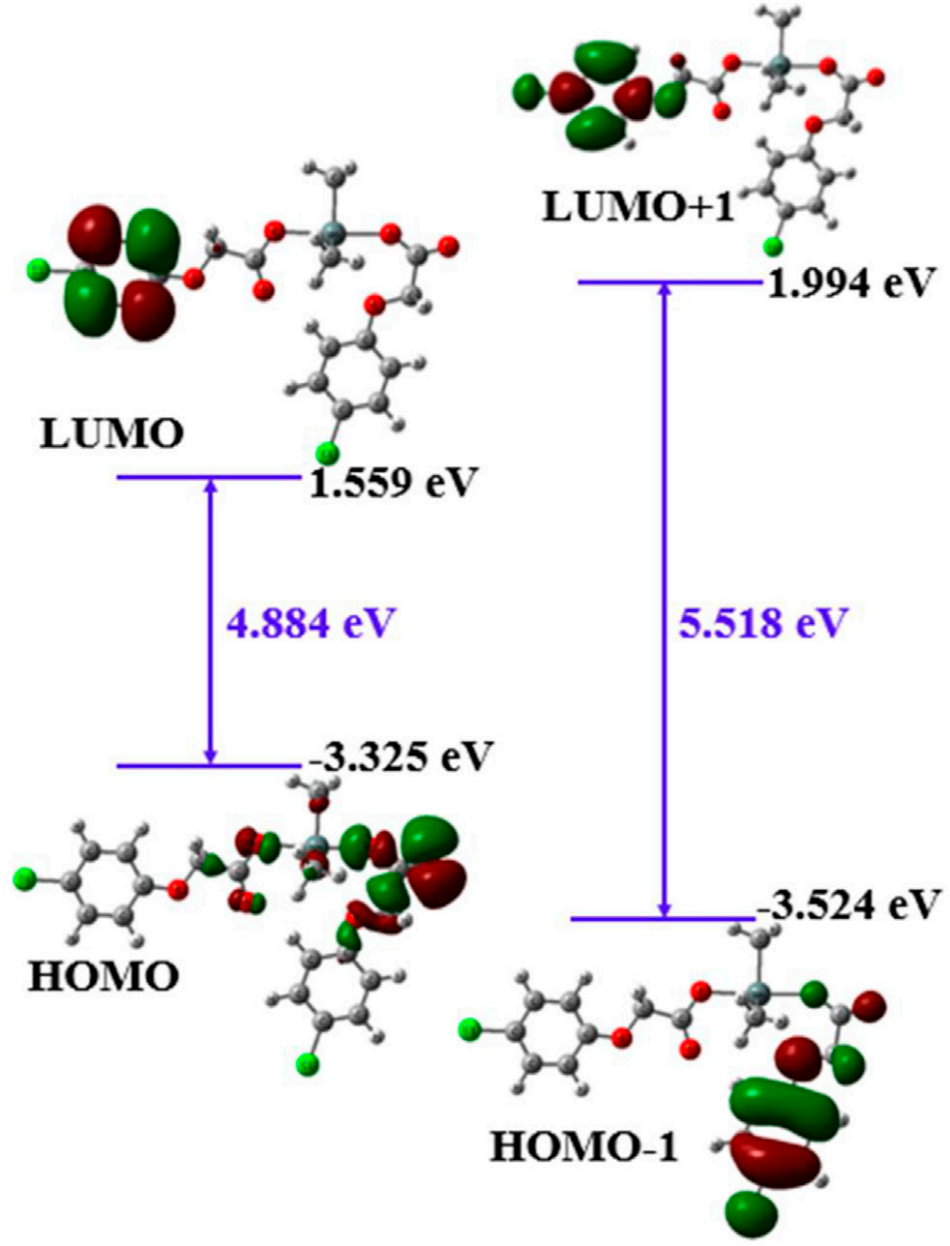
The FMOs of complex **4** determined at B3LYP/6-31G* + LANL2DZ method.

The Frontier molecular orbitals (FMOs) play an essential role in defining the intramolecular charge transfer properties, reactivity and optical properties of a complex. The FMOs of complex **4** are depicted in Figure 4, which include HOMO-1, HOMO, LUMO, LUMO + 1 orbitals along with their orbital energies and orbital energy gaps (E_g_). In metal complexes, there are different types of intramolecular charge transfer processes including metal to ligands, ligand to metal and ligand to ligand, etc. A careful analysis of Figure 4 shows that there is ligand to ligand intramolecular charge transfer upon electronic transition. It can be visualized from Figure 4 that the HOMO is localized mainly on the methoxy acetate groups of complex **4**, while LUMO is confined to chlorobenzene ring of one side ligand. There is very little and/or negligible electron density on Sn metal which indicates that the Sn metal acts as more like a bridge for intramolecular charge transfer or redistribution upon electron transitions. On the other hand, the distributions of HOMO-1 to LUMO + 1 orbitals solely involve the chlorobenzene rings with significant contributions from chlorine atoms as can be seen in Figure 4. Overall a ligand to ligand charge transfer is evident from the analysis of Frontier molecular orbital analysis which indicates that the optoelectronic properties of complex **4** might be tuned exclusively by the ancillary ligands (Vogler and Kunkely, 1997).

#### 3.4.4 *In Vitro* Cytotoxicity of Ligand Acid HL and Complexes 1–4

**FIGURE 5:**
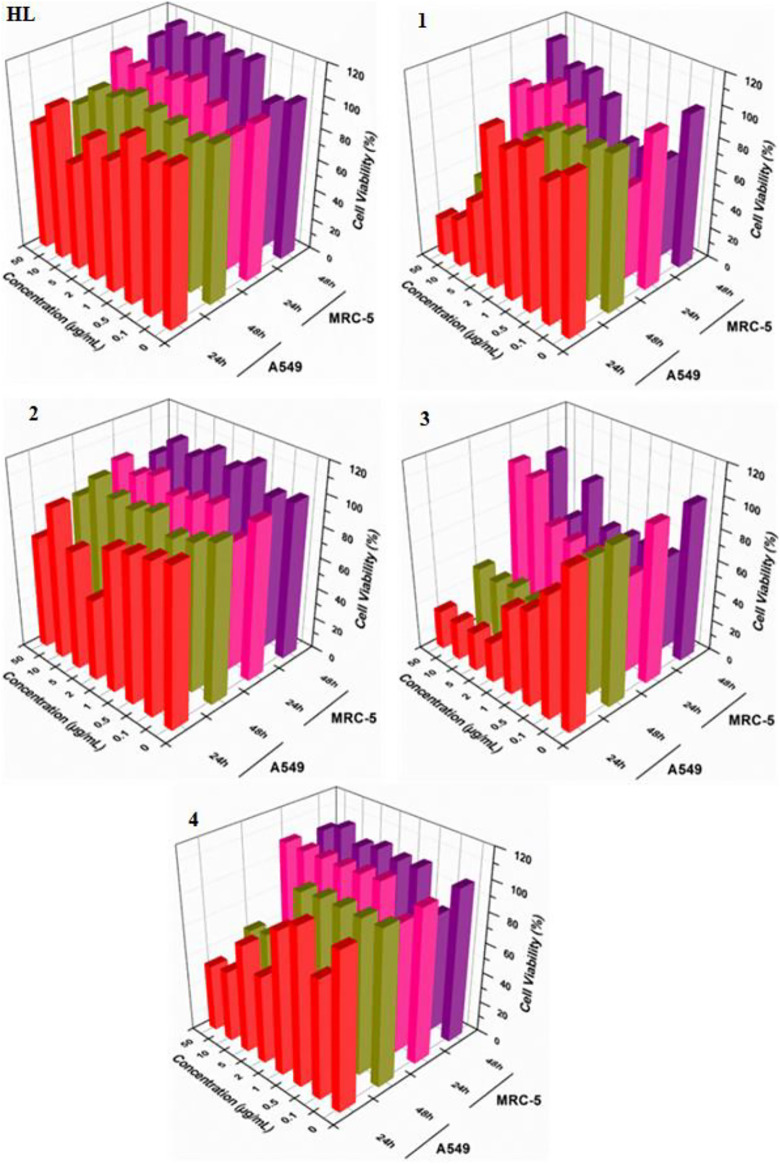
Cytotoxic effects of ligand acid (**HL**) and synthesized complexes (**1–4**) against A549 and MRC-5.

**FIGURE 6:**
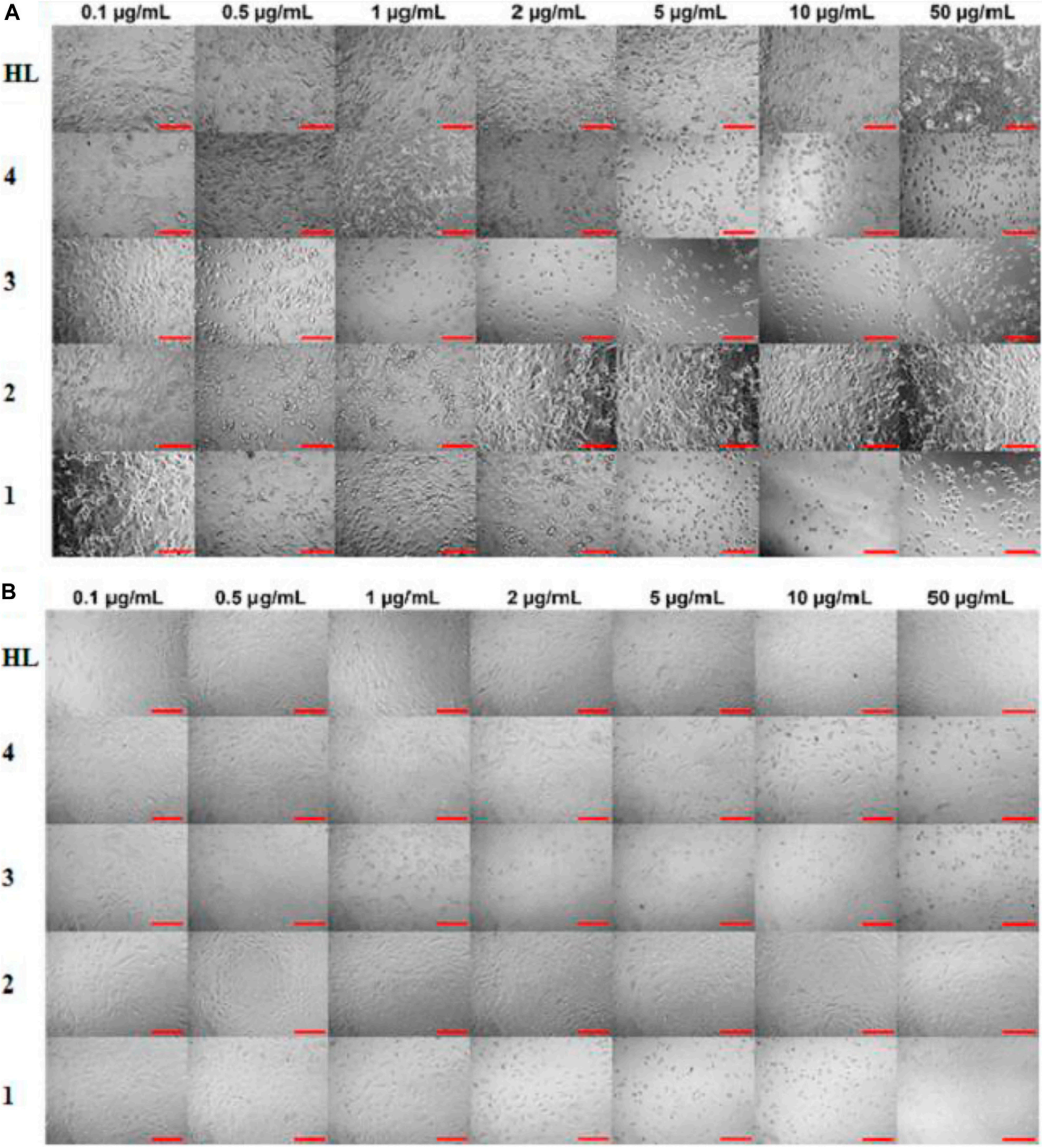
Effect of ligand acid (**HL**) and complexes **1–4** on morphology of **(A)** A549 and **(B)** MRC-5 for 48 h. The scale bar is 100 µm.

*In vitro* cytotoxic effect of the free ligand acid **HL** and synthesized complexes **1–4** was assessed against A549 and MRC-5 at different concentrations (0, 0.1, 0.5, 1, 2, 5, 10 and 50 μg/ml) for 24 and 48 h. The amount of DMSO used to dissolve the compounds was applied below the cytotoxicity limit (less than 0.1% range as suggested for the cell culture). The experimental findings regarding cell viability are presented in Figure 5 as well in tabulated form in Supplementary Table 1. Also, Figure 6 show the effect of studied concentrations of the tested compounds on the morphology of A549 and MRC-5 cell lines. The IC_50_ values are given in Table 3.

**TABLE 3:** IC_50_ values (µg/ml) of the selected compounds.

Compound #	A549		MRC5	
	24 H	48 H	24 H	48 H
HL	58.97316	NC^a^	NC	NC
1	44.41323	NC	NC	NC
2	15.38864	16.65308	NC	NC
3	13.46048	17.10775	NC	NC
4	19.77546	22.04041	NC	NC

a NC: not calculated.

All the tested compounds showed an anticancer activity against A549 cell line. In a dose dependent manner, the anticancer activity of complexes **1**, **3** and **4**, increased by increasing their concentrations at 24 h. These complexes (**1**, **3** and **4**) were found particularly more active anticancer agents against A549 at higher concentrations. Interestingly, these complexes are not toxic at higher concentrations against MRC-5 cell line. However, complexes **1** and **3** exhibited cytotoxicity against MRC-5 at lower concentrations. The anticancer activity of the complexes **1**, **3** and **4** slightly decreased after 48 h. The free ligand acid **HL** and complex **2** showed a lower and stable anticancer activity against A549 at all studied concentrations, at both 24 and 48 h. Also the toxic effect of the free ligand acid **HL** and complex **2** against MRC-5 was relatively very low at all studied concentrations.

The experimental results suggested a metal-based activity, as complexation increased the anticancer activity of the free ligand acid **HL**. The activity was also affected by the length of the alkyl group, as complexes **1** and **3** with bulky *n*-butyl groups were found more active than complexes **2** and **4** with smaller methyl groups. In fact, among the tested complexes, the complex **3** showed higher anticancer activity. This reflects the importance of both lipophilicity as well as tin coordination number in anticancer activity of the synthesized complexes. In addition, the presence of three nonpolar *n*-butyl groups in complex **3** makes it more lipophilic. So, the movement of the complex through the cellular membrane of the target cell to reach the action site is easy. Also, the low coordinated tin atom (coordination number = 4 as confirmed by NMR spectra) in complex **3** is more exposed for interaction with the donor atoms of the biomolecule of the target cell. The argument is further supported by the lower cytotoxicity of complex **2**, where, in this complex two smaller methyl groups and a higher coordination number of six as confirmed by NMR spectra offer lower lipophilicity and lesser exposed tin center, respectively. This makes the complex **2** a relatively weaker anticancer agent.

#### 3.4.5 Antibacterial Activity

Table 4 describes the antibacterial activity of the ligand acid (**HL**) and synthesized complexes **1–4**, assessed against five bacterial strains namely *E. coli, S. aureus*, *P. aeregionosa*, *K. pneumonia* and *B. cereus*. The nature of the bacterial strain seems to be the dominant factor affecting the antibacterial efficiency of the tested compounds. Generally the activity of the complexes **1–4** is higher than that of the free ligand acid (**HL**). Comparing the activity of the complexes **1–4** with the standards, Streptomycin and Ampicillin, it can be seen from the data their activity is very slightly smaller than the standards against the tested strains. Among the tested complexes, *n*-butyltin (IV) derivatives (**1** and **3**) are more active than methyltin (IV) derivatives (**2** and **4**). The bulky *n*-butyl groups offer more lipophilic (organic) character to the complexes and thus making them more active in the present study.

**TABLE 4:** *In vitro* antibacterial activity of the screened compounds.

	Zone of inhibition (mm)				
Compound	*E. coli*	*S. aures*	*P. aeregionosa*	*K. pneumoniae*	*B. cereus*
1	16.58 ± 0.87	15.08 ± 1.28	15.66 ± 1.01	15.75 ± 0.90	15.08 ± 0.94
2	17.41 ± 0.62	17.75 ± 0.90	16.75 ± 0.25	17.41 ± 0.94	16.75 ± 1.75
3	16.25 ± 0.66	15.33 ± 1.23	16.16 ± 0.52	16.41 ± 0.80	17.08 ± 0.38
4	16.08 ± 0.57	15.58 ± 1.12	14.66 ± 1.01	15.58 ± 0.38	15.5 ± 0.87
HL	14.91 ± 0.52	15.08 ± 0.94	14.5 ± 0.86	14.75 ± 0.75	15.08 ± 0.38
DMSO	—	—	—	—	—
Streptomycin	17.91 ± 0.14.	Un-checked	Un-checked	18.5 ± 0.5	17.91 ± 0.87
Ampicillin	Un-Checked	18.41 ± 0.41	18.16 ± 0.52	Un-checked	Un-checked

### 3.5 Molecular Docking Studies

#### 3.5.1 Complex-DNA/Protein Interaction

**FIGURE 7:**
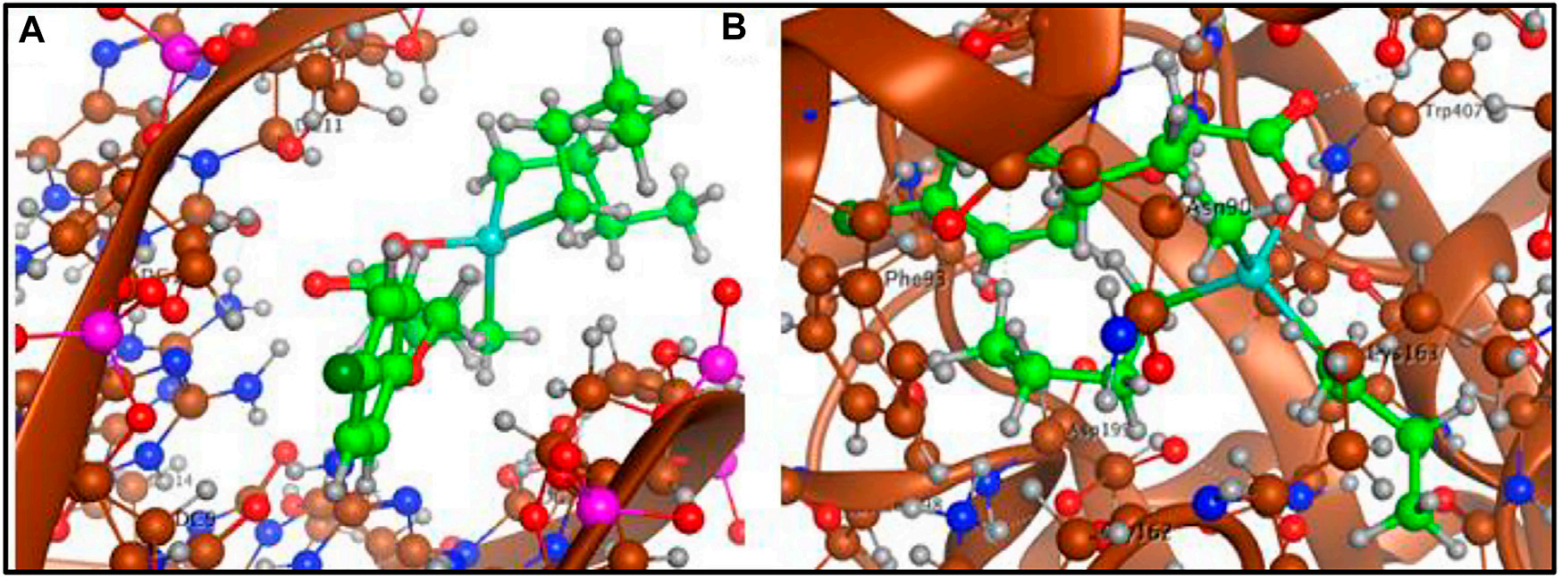
**(A)** Complex **3**-DNA interaction and **(B)** Complex **3**-tubulin interaction.

To have a theoretical insight about the anticancer activity of the synthesized complexes, the DNA interactions were performed for the most active complex **3**. The complex **3** has shown interaction with DNA in form of intercalation. The complex **3** show four polar interactions (docking score = −3.6005), two H-donor with DG 16, DA 17 and two π-H with DC 9, DG 10 active sites of DNA as shown in Figure 7A.

The interacting ability of the complex **3** was also assessed with tubulin (PDB ID 1SA0) in complex with colchicine as a target protein structure. Complex **3** was stabilized in the colchicine binding cavity by three polar interactions (docking score −5.2957), one H-donor with LEU 195, one H- acceptor with TRP 407 and one H-π with PHE 93 active sites of the tubulin as shown in Figure 7B. The docking analysis has shown that complex **3** offers best dock pose with tubulin in colchicine binding cavity compared to DNA.

#### 3.5.2 SARS-CoV-2 Interaction

**FIGURE 8:**
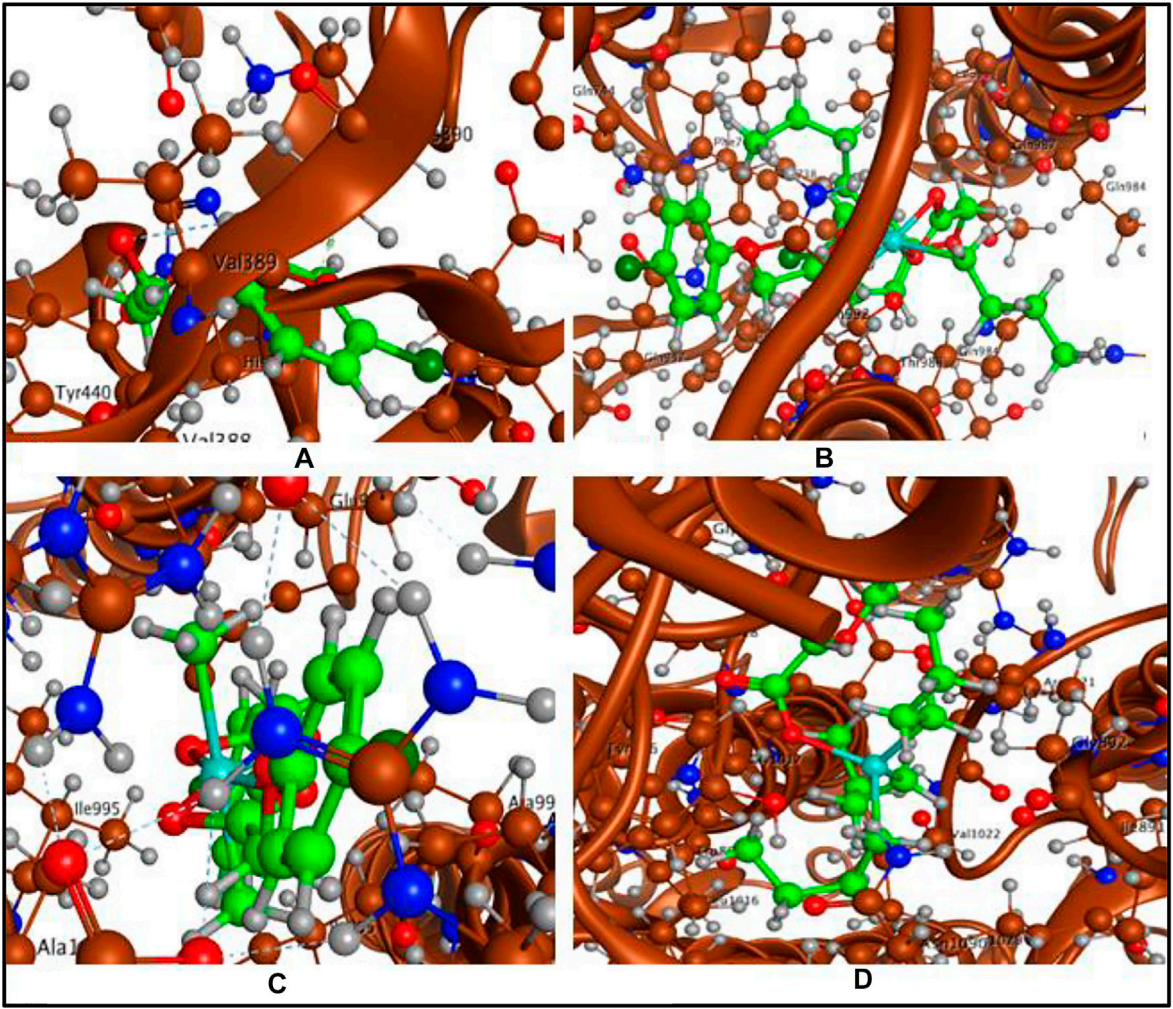
**(A)** Ligand acid (**HL**)-spike protein interaction, **(B)** Complex **1**-spike protein interaction, **(C)** Complex **2**-spike protein interaction and **(D)** Complex **3**-spike protein interaction.

The ligand acid and its organotin(IV) derivatives were docked with spike protein, nucleocapsid protein of corona virus SARS-CoV-2 and angiotensin converting enzyme (ACE2) of human. The docking analysis have shown reasonable interactions of the screened compounds with the active sites of the targets. The ligand acid **HL** (docking score = −3.6103) has shown four polar interactions, two H-acceptor, one π-H and one H-donor interaction with VAL 389, LYS 390, LYS 390 and GLU 37 active sites residues of spike protein as shown in Figure 8A. Complex **1** (docking score = −7.6506**)** has best docked pose with three polar H-donor interactions with SER 985, GLY 981 active residues of the spike protein as shown in Figure 8B. Complex **2** (docking score = −5.1539) has shown two polar interactions, one H-acceptor and one π-H with ILE 995 and ALA 998 active residues of spike protein as shown in Figure 8C. Complex **3** (docking score = −5.5353) has shown three polar interactions, two are H-π and one are H-acceptor with TRP 868, TYR 1029 and LYS 1020 active residues of spike protein as shown in Figure 8D. Complex **4** (docking score = −3.7082) has shown three polar interactions, one H-donor, one H-acceptor and one π-π interaction with LYS 1020, TRP 868 and TYR 1029 active residues of the spike protein. These polar interactions reflect that the synthesized complexes can interact spike protein and affect its ability to develop any interaction with the host receptor.

**FIGURE 9:**
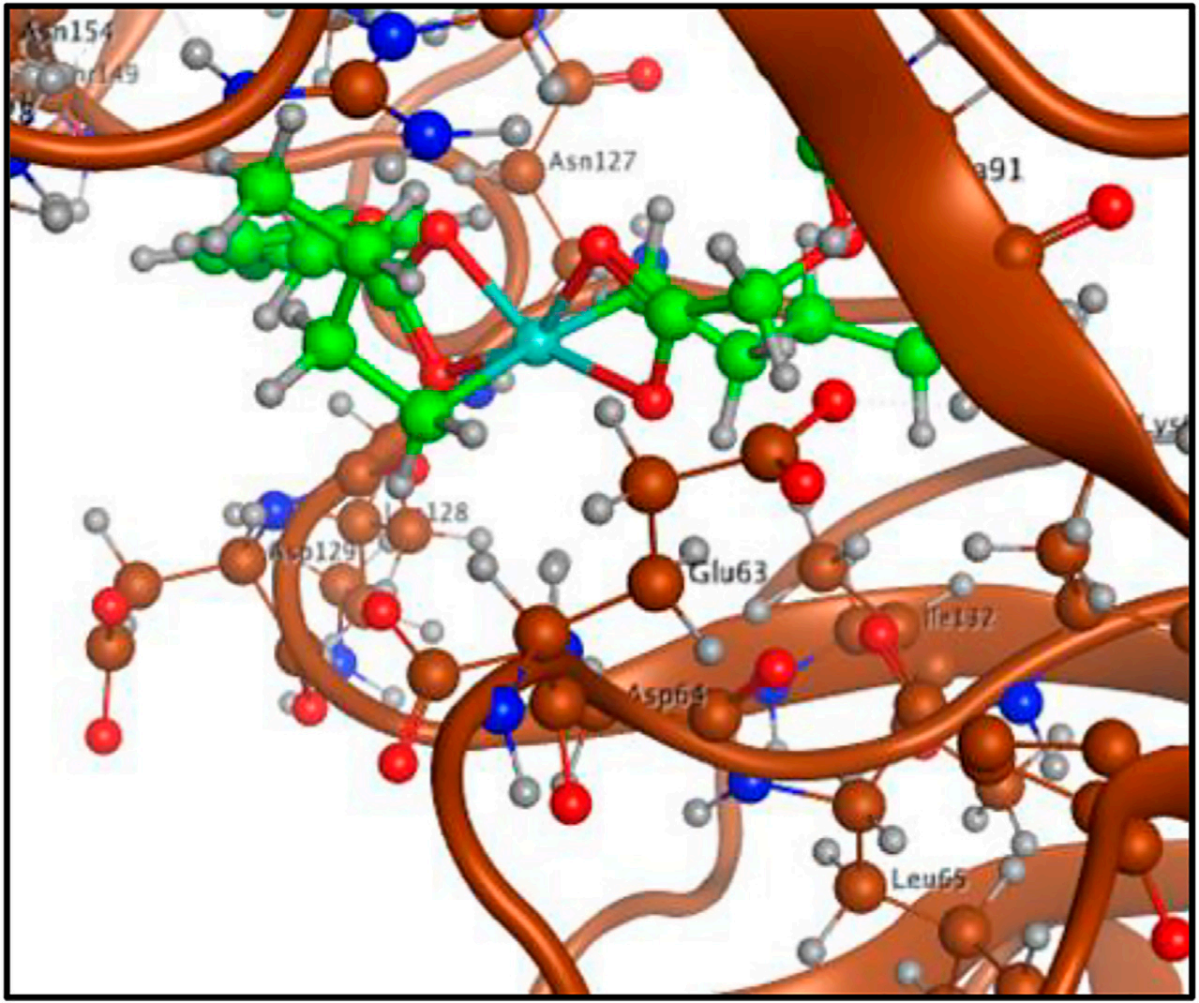
Complex **1**-nucleocapsid protein interaction.

The ligand acid and synthesized complexes have also shown interactions with the nucleocapsid protein of the corona virus SARS-CoV-2. Complex **1** has shown best dock pose (−5.7402) with two polar interactions, one H-donor and one π-H interaction with GLU 63 and TRP 53 active site residues, respectively of the nucleocapsid protein as shown in Figure 9. The ligand acid **HL** (−3.2769), complex **2** (−3.1275), complex **3** (−3.6005) and complex **4** (−2.2132) have shown three, two, four and two polar interactions, respectively in form of H-donor/acceptor, π-H and H-π polar interactions as mentioned in Table 5 (Supplementary Figures 10–13). The higher docking scores of the test compounds with spike protein compared to the nucleocapsid protein show that the test compounds offer best dock poses for interactions with spike protein as target for the corona virus SARS-CoV-2 inhibition.

**TABLE 5:** Ligand acid (**HL**) and complexes **1**–**4** interaction report with nucleocapsid protein.

Code	Ligand	Receptor	Residue	Interaction	Distance	E	Docking score
HL	C 6	O	ARG 69	(B) H-donor	3.57	−0.2	−3.2769
	O 17	OH	TYR 173	(C) H-acceptor	2.84	−1.1	
	6-ring	CB	LEU 160	(C) pi-H	4.67	−0.2	
1	C 11	OE1	GLU 63	(B) H-donor	2.98	−0.2	−5.7402
	6-ring	CH2	TRP 53	(D) pi-H	4.30	−0.2	
2	Cl 27	O	PHE 54	(C) H-donor	3.31	−0.9	−3.1275
	6-ring	CB	SER 106	(A) pi-H	4.85	−0.2	
3	Cl 28	NE2	HIS 60	(C) H-acceptor	3.39	−0.3	−3.6005
	C 24	6-ring	TYR 173	(C) H-pi	4.04	−0.4	
	6-ring	CG2	THR 58	(A) pi-H	4.93	−0.2	
	6-ring	CG2	THR 58	(C) pi-H	4.88	−0.2	
	6-ring	CG2	THR 58	(C) pi-H	4.88	−0.2	
4	Cl 31	O	PHE 54	(C) H-donor	3.44	−0.6	−2.2132
	C 2	6-ring	TYR 173	(C) H-pi	4.01	−0.5	

**FIGURE 10:**
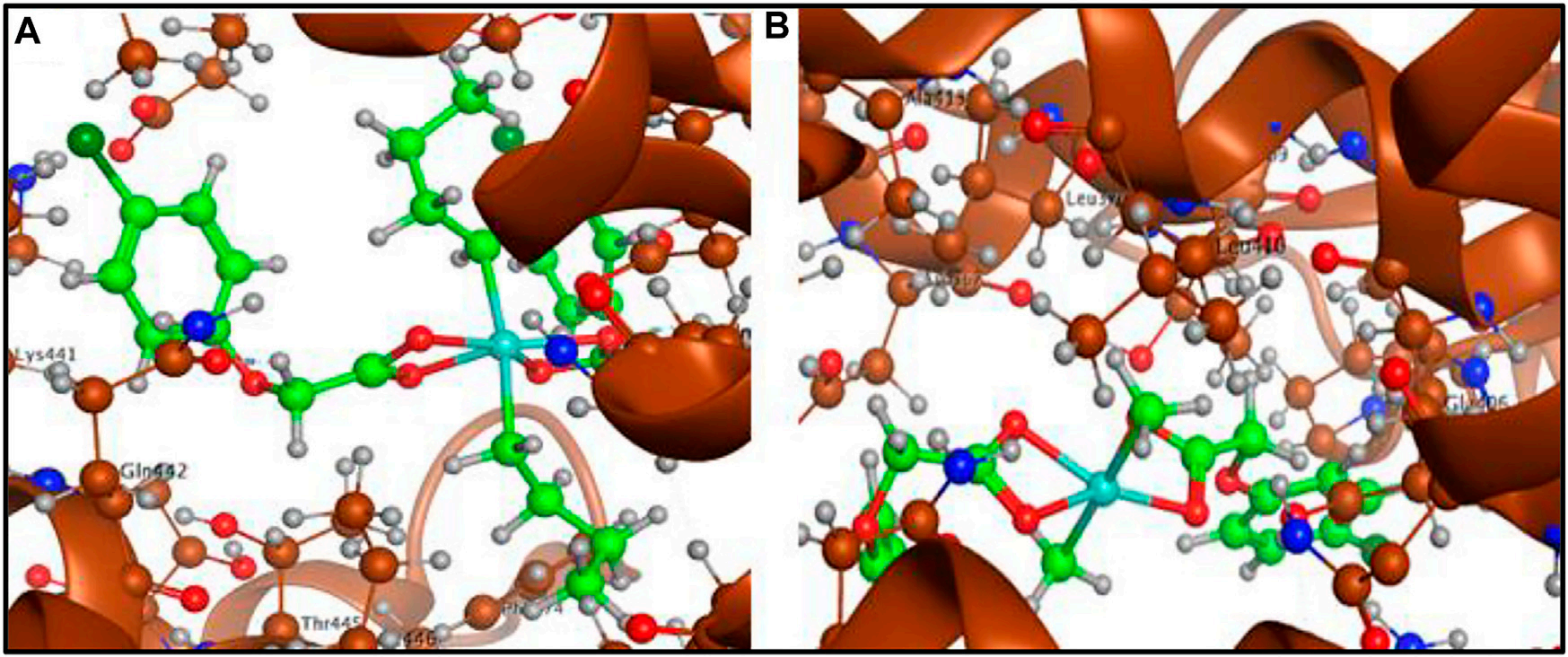
**(A)** Complex **1**-angiotensin converting enzyme interaction and **(B)** Complex **2**-angiotensin converting enzyme interaction.

Molecular docking studies with angiotensin converting enzyme also reflected best dock pose for the complex **1** (docking score = −6.1678) with four polar interactions, one H-donor and three H-acceptor interactions with GLN 442, PHE 438, ARG 518 and THR 371 active sites as shown in Figure 10 (a). Complex **2** (docking score = −5.6760) has shown six polar interactions, two H-donor and one H-π and three π-H interactions with GLU 406, ASP 367, HIS 374, THR 371, and LYS 441 active sites as shown in Figure 10B. Complex **3** (docking score = −5.1188), complex **4** (docking score **=** −3.5105**)** and ligand acid **HL** (docking score = −3.4990) have shown one, three and two polar interactions respectively, with the active site residues of the angiotensin converting enzyme as mentioned in Table 6 (Supplementary Figures 14–17). The docking analysis shows that the free ligand acid and its complexes can interact with the three possible targets sites involved in corona disease. The complex **1** with relatively higher docking scores offered the best dock poses for the interaction with the active site residues of all the three targets.

**TABLE 6:** Ligand acid (**HL**) and complexes **1–4** interaction report with angiotensin converting enzyme (ACE2).

Code	Ligand	Receptor	Residue	Interaction	Distance	E	Docking score
HL	C 8	O	PRO 490	(A) H-donor	3.25	−0.3	−3.4990
	6-ring	CB	TYR 613	(A) pi-H	3.52	−0.6	
1	C 11	OE1	GLN 442	(A) H-donor	3.05	−2.0	−6.1678
	Cl 25	CE1	PHE 438	(A) H-acceptor	3.68	−0.2	
	O 48	NH2	ARG 518	(A) H-acceptor	2.77	−0.6	
	Cl 63	CG2	THR 371	(A) H-acceptor	3.93	−0.2	
2	C 13	OE2	GLU 406	(A) H-donor	3.35	−0.4	−5.6760
	C 36	OD1	ASP 367	(A) H-donor	3.16	−0.2	
	C 25	5-ring	HIS 374	(A) H-pi	3.68	−0.6	
	6-ring	CG2	THR 371	(A) pi-H	3.63	−0.2	
	6-ring	CG	LYS 441	(A) pi-H	4.34	−0.4	
	6-ring	CE	LYS 441	(A) pi-H	4.03	−0.3	
3	Cl 28	OH	TYR 515	(A) H-acceptor	3.59	−0.2	−5.1188
4	Cl 31	CE	MET 366	(A) H-acceptor	3.58	−0.2	−3.5105
	Cl 31	CE1	PHE 438	(A) H-acceptor	3.57	−0.2	
	Cl 31	NZ	LYS 441	(A) H-acceptor	3.41	−0.9	

## 4 Conclusion

Four organotin(IV) derivatives (*n*-C_4_H_9_)_2_SnL_2_ (**1**), (CH_3_)_2_SnL_2_ (**2**), (*n*-C_4_H_9_)_3_SnL (**3**) and (CH_3_)_3_SnL (**4**) of 4-chlorophenoxyacetic acid (**HL**) were synthesized and characterized. The ligand has shown a chelating (**1** and **2**) and bridging (**3** and **4**) coordination modes to the tin centre in solid state. The NMR (^1^H, ^13^C and ^119^Sn) results have shown higher coordination number for tin in diorganotin(IV) derivatives (**1** and **2**) compared to the triorganotin(IV) derivatives (**3** and **4**) in solution. The calculated structure of complex **4** at B3LYP/6-31G* + LANL2DZ level of theory indicated a good resemblance with the experimentally determined X-ray single crystal structure. Quantum chemical analysis indicated the significant involvement of the 4-chlorophenoxyacetate in the formation of Frontier molecular orbitals of the complex **4**. The *in vitro* cytotoxic effect of the **HL** against A549 and MRC-5 has increased after complexation with organotin(IV) moieties in the complexes **1–4**. Complex **3** with more lipophilic character and low coordinated tin atom offered higher cytotoxicity. The *in vitro* antibacterial activity order **HL** > *n*-butyltin(IV) derivatives (**1** and **3**) > methyltin(IV) deivaitves (**2** and **4**) against the tested bacterial strains reflected an organic mode of action for the tested compounds. The docking study (for complex **3**) has suggested tubulin in complex with colchicine (docking score −5.2957) as better anticancer target compared to DNA (docking score = −3.6005). The docking interactions of the synthesized complexes (especially **1)** with all the three possible targets (spike and nucleocapsid protein of corona virus SARS-CoV-2 and angiotensin converting enzyme (ACE2) of human) make the organotin(IV) carboxylates as potential candidates for further studies in corona virus treatment in future.

## Data Availability

The datasets presented in this study can be found in online repositories. The names of the repository/repositories and accession number(s) can be found in the article/Supplementary Material.
