# Supplementary material for: Synthesis, Characterization, Biological Activity and Molecular Docking Studies of Novel Organotin(IV) Carboxylates
Source: Front Pharmacol. 2022 Apr 5;13:864336. doi: 10.3389/fphar.2022.864336 (PMC9017761; doi:10.3389/fphar.2022.864336)
Supplement: Supplementary file 1 [file DataSheet2.docx]

Supplementary Material


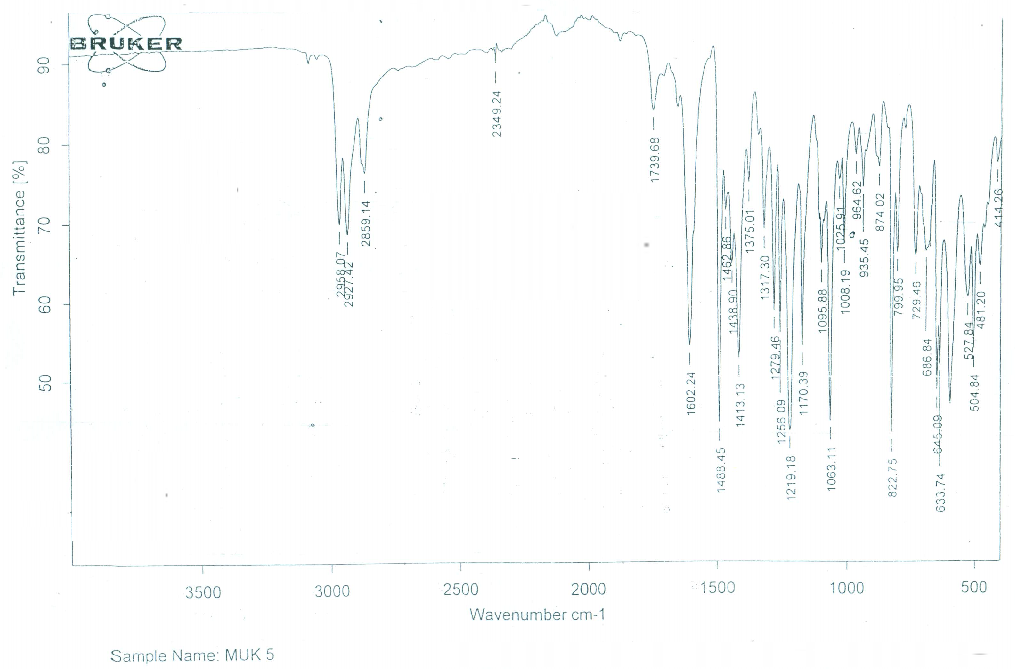


**Supplementary Figure 1.** FT-IR spectrum of complex **1**


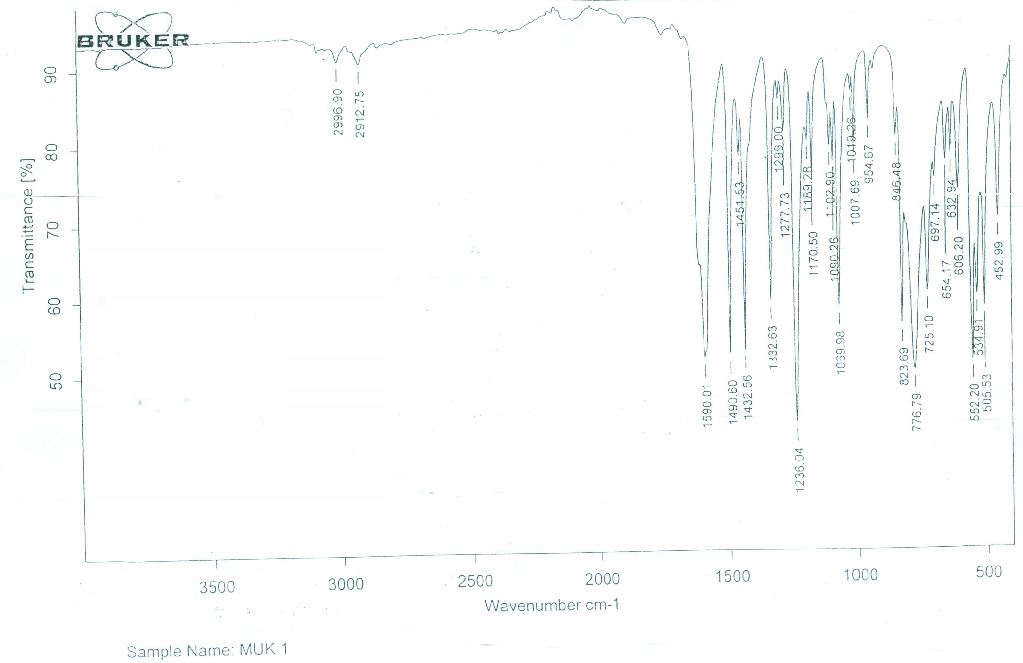


**Supplementary Figure 2.** FT-IR spectrum of complex **4**


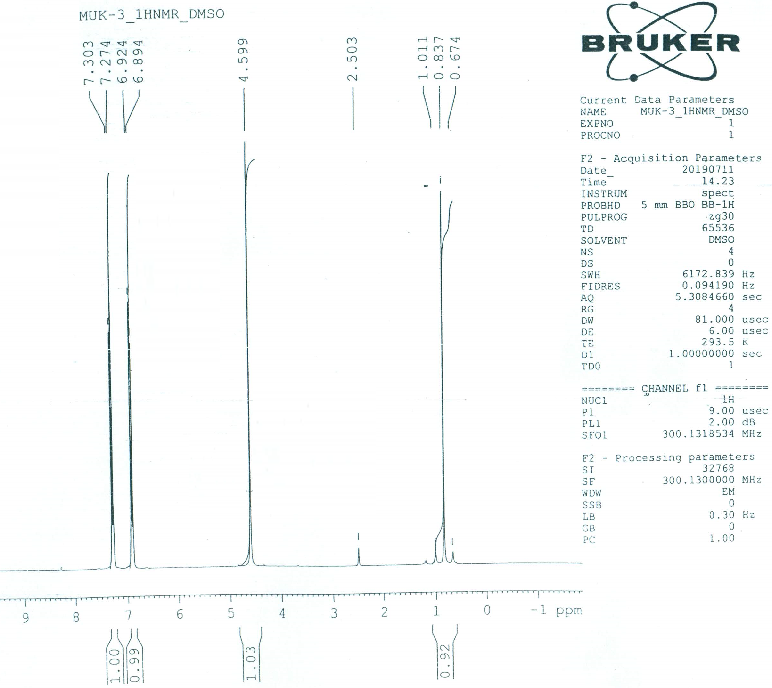


**Supplementary Figure 3.** ^1^H spectrum of complex **2**


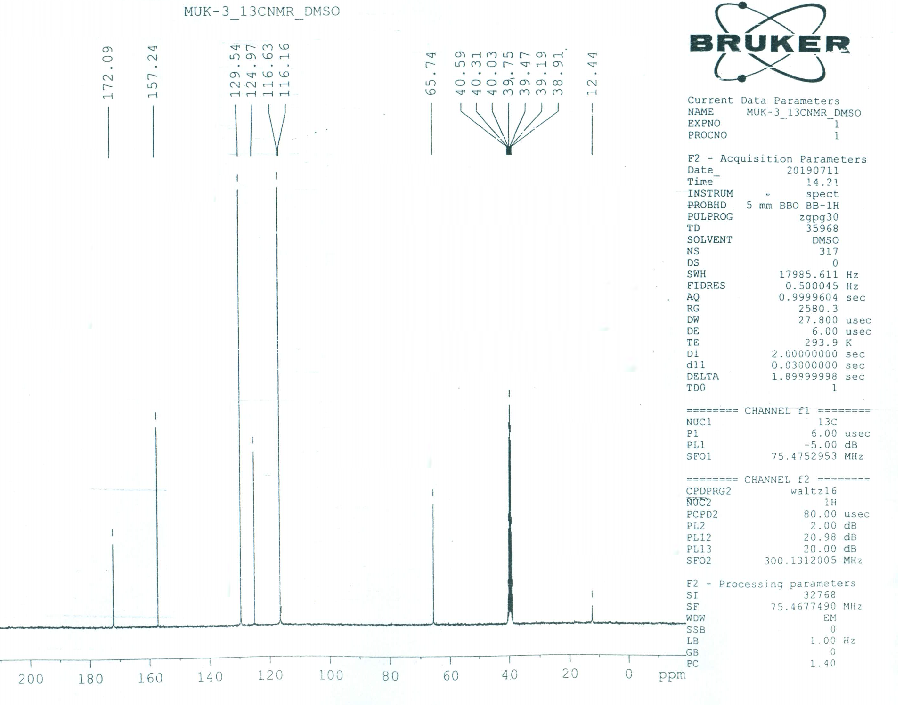


**Supplementary Figure 4.** ^13^C spectrum of complex **2**


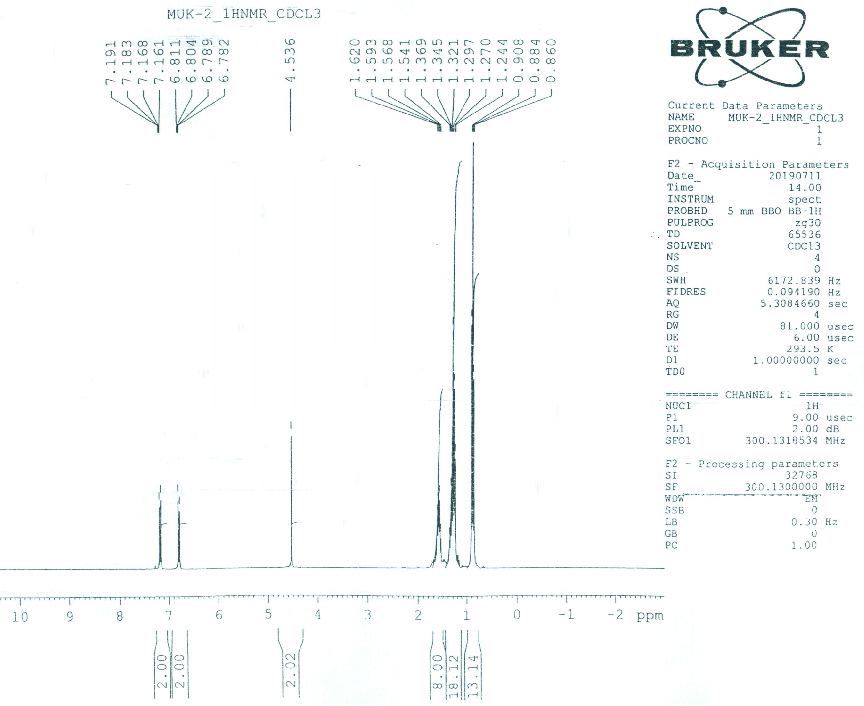


**Supplementary Figure 5.** ^1^H spectrum of complex **3**


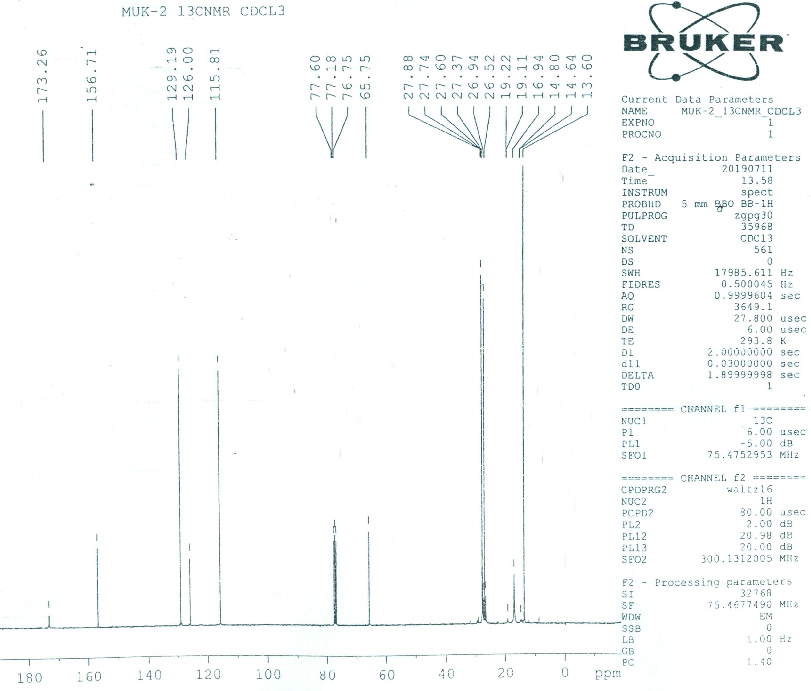


**Supplementary Figure 6.** ^13^C spectrum of complex **3**


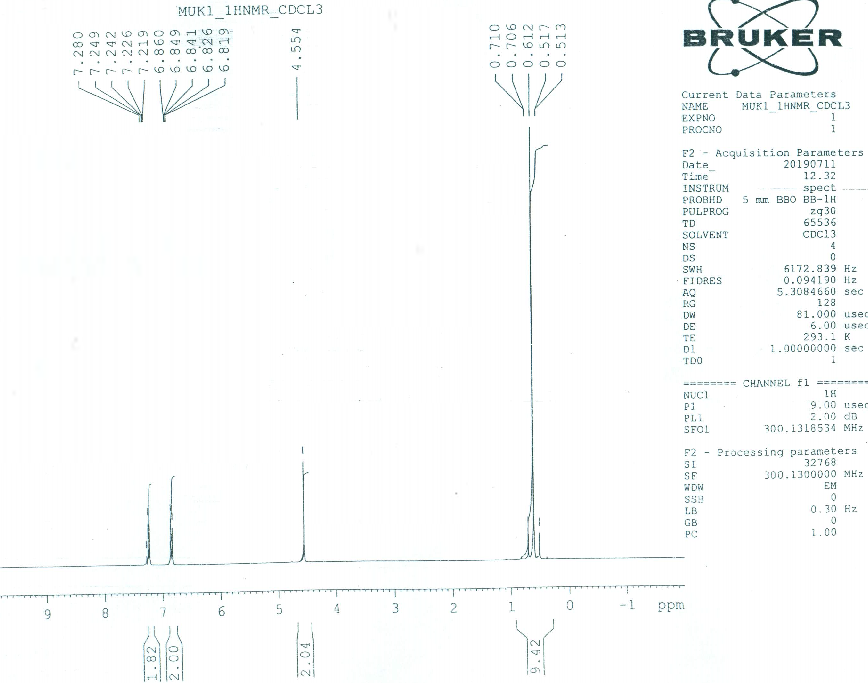


**Supplementary Figure 7.** ^1^H spectrum of complex **4**


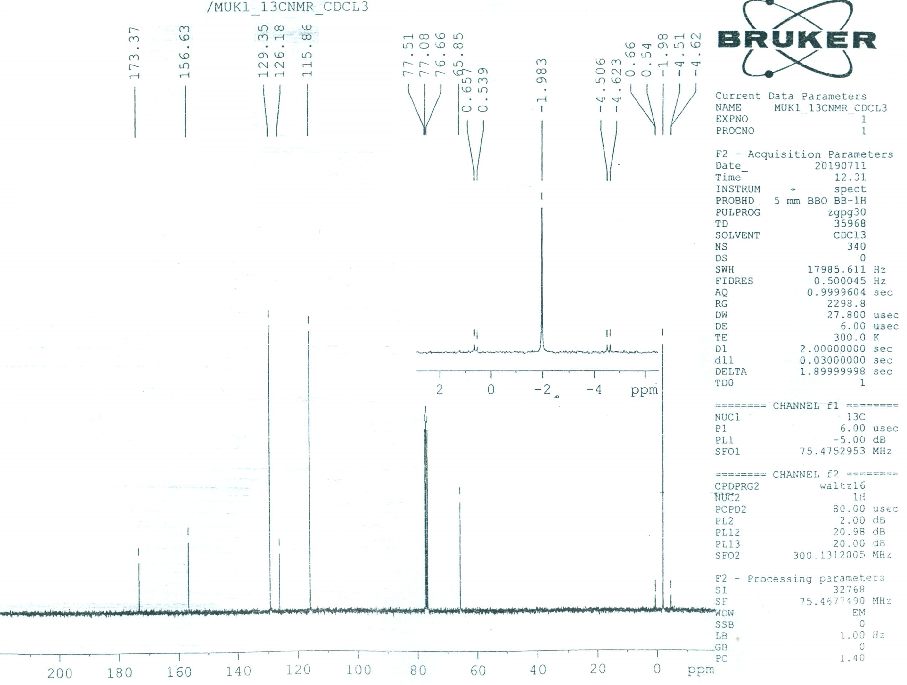


**Supplementary Figure 8.** ^13^C spectrum of complex **4**


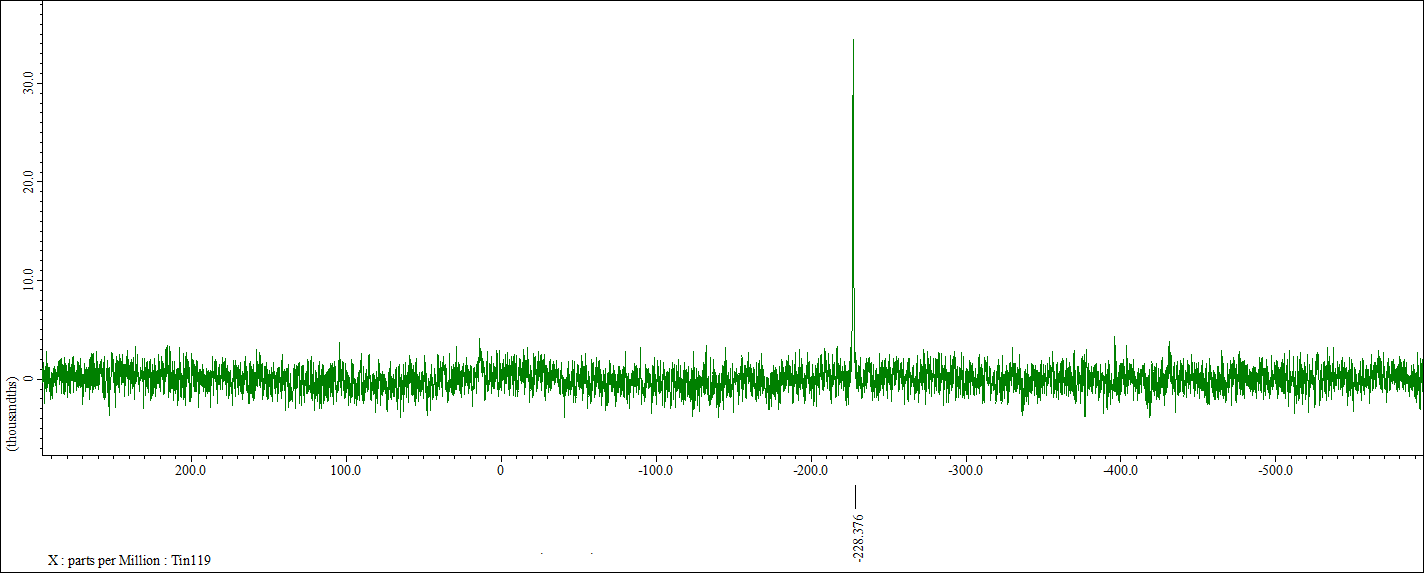


**Supplementary Figure 9.** ^119^Sn spectrum of complex **1**

**Optimized coordinates of complex 4 at B3LYP/6-31G*+LANL2DZ level of theory**

Cl -2.48014800 6.11434400 -0.00023300

O -6.06067900 -2.24279500 0.00045300

O -3.82191300 -2.44527900 -0.00011800

O -3.55553700 0.29549100 0.00058100

C -4.92104600 -1.77841400 0.00026700

C -4.88013500 -0.23187900 0.00038700

H -5.42535900 0.11584500 -0.88687400

H -5.42559900 0.11571300 0.88755000

C -3.38167200 1.64492800 0.00037900

C -2.04956200 2.09688700 0.00028000

H -1.23414800 1.37733100 0.00033000

C -1.77727400 3.45994900 0.00009600

H -0.74830900 3.80409100 0.00000900

C -2.83057400 4.37798300 0.00001400

C -4.15098500 3.94622800 0.00012200

H -4.96241500 4.66710800 0.00006900

C -4.43067300 2.57560600 0.00030900

H -5.46401300 2.24981600 0.00042000

Sn -1.66636500 -2.17744100 -0.00020300

Cl 9.28170100 0.57336600 0.00037400

O 0.56093500 -2.00110700 -0.00054100

O 0.69956100 0.26804200 0.00006700

O 3.38403900 0.16490700 -0.00040400

C 1.17328700 -0.86841700 -0.00022200

C 2.69960800 -1.08532700 -0.00009700

H 2.97160900 -1.67187900 0.88758300

H 2.97172200 -1.67240200 -0.88739000

C 4.73439900 0.16851500 -0.00020200

C 5.34235800 1.43807500 -0.00049900

H 4.69987700 2.31279700 -0.00086200

C 6.72516800 1.56500600 -0.00032400

H 7.18674500 2.54715500 -0.00055400

C 7.52033700 0.41577800 0.00015100

C 6.94045100 -0.84744700 0.00044600

H 7.56656900 -1.73394900 0.00080800

C 5.54809700 -0.97557800 0.00026900

H 5.10987200 -1.96638500 0.00049000

C -1.65724400 -1.31194500 1.95867700

H -1.05657400 -1.94812800 2.61904600

H -2.67140900 -1.23898900 2.35895400

H -1.20032400 -0.32015800 1.93323300

C -1.65782000 -1.31107700 -1.95870400

H -2.67211400 -1.23781800 -2.35858600

H -1.05743700 -1.94698500 -2.61959800

H -1.20077200 -0.31935300 -1.93293700

C -1.45730700 -4.31108600 -0.00027100

H -0.39955000 -4.59134800 0.00014700

H -1.94176200 -4.74124500 -0.88332900

H -1.94249200 -4.74100200 0.88247400

**
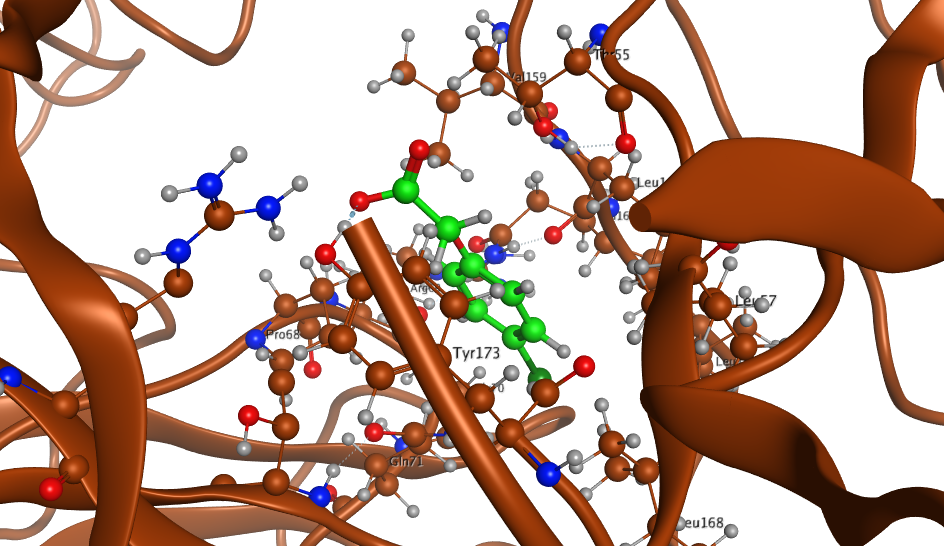
**

**Supplementary Figure 10.** Ligand acid (**HL**) interaction with nucleocapsid protein.

**
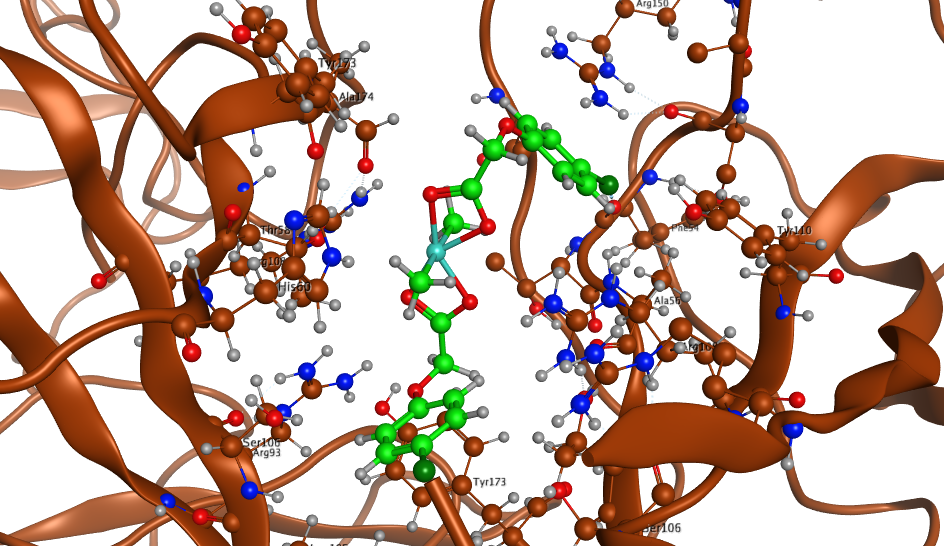
**

**Supplementary Figure 11.** Complex **2** interaction with nucleocapsid protein.

**
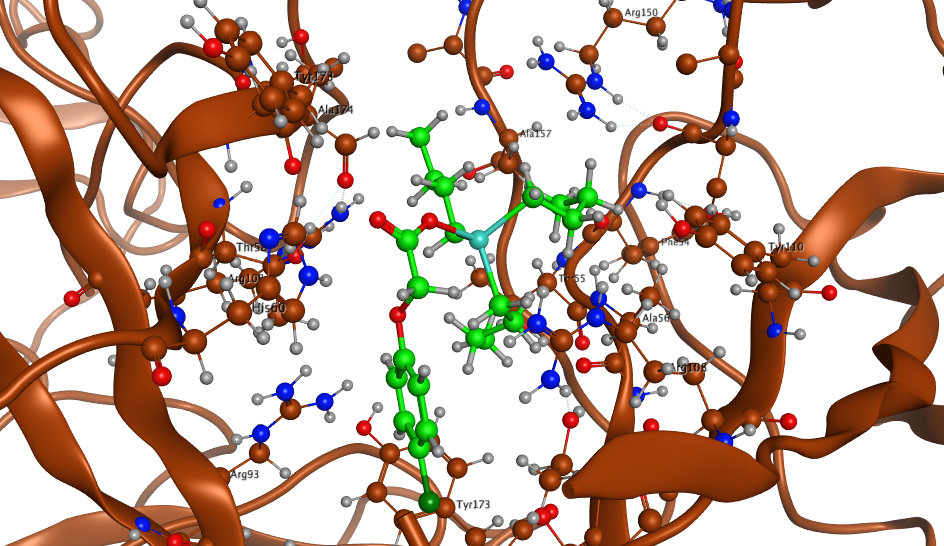
**

**Supplementary Figure 12.** Complex **3** interaction with nucleocapsid protein.

**
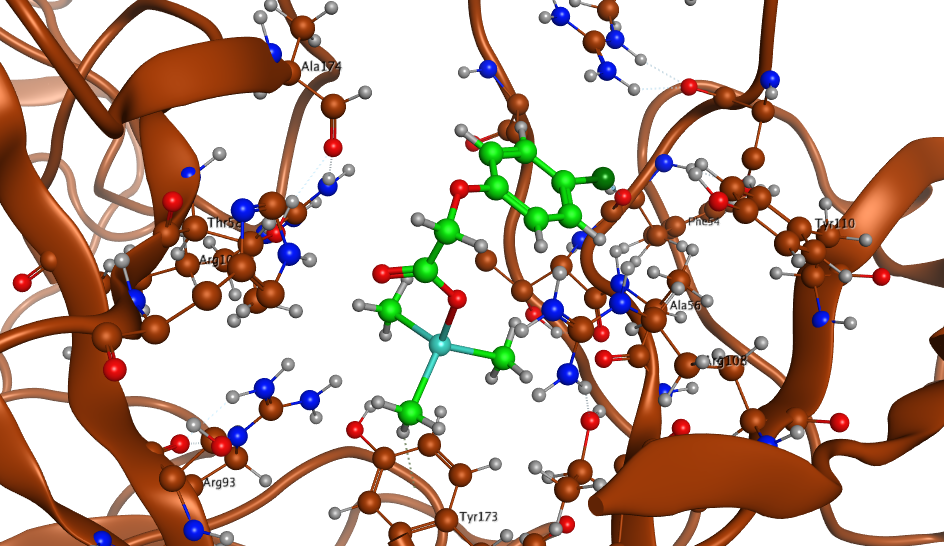
**

**Supplementary Figure 13.** Complex **4** interaction with nucleocapsid protein.


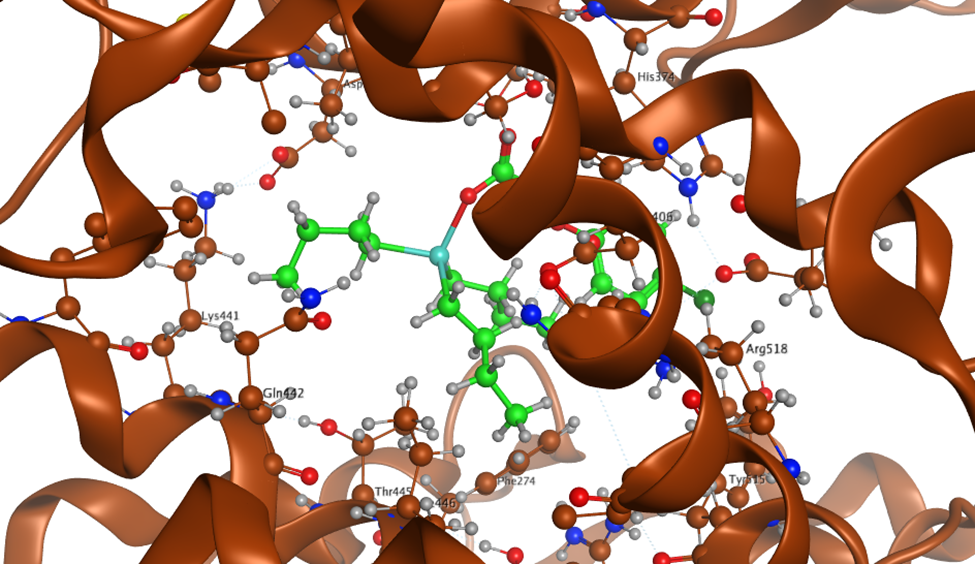


**Supplementary Figure 14.** Complex **3** interaction with angiotensin converting enzyme (ACE2) of human.


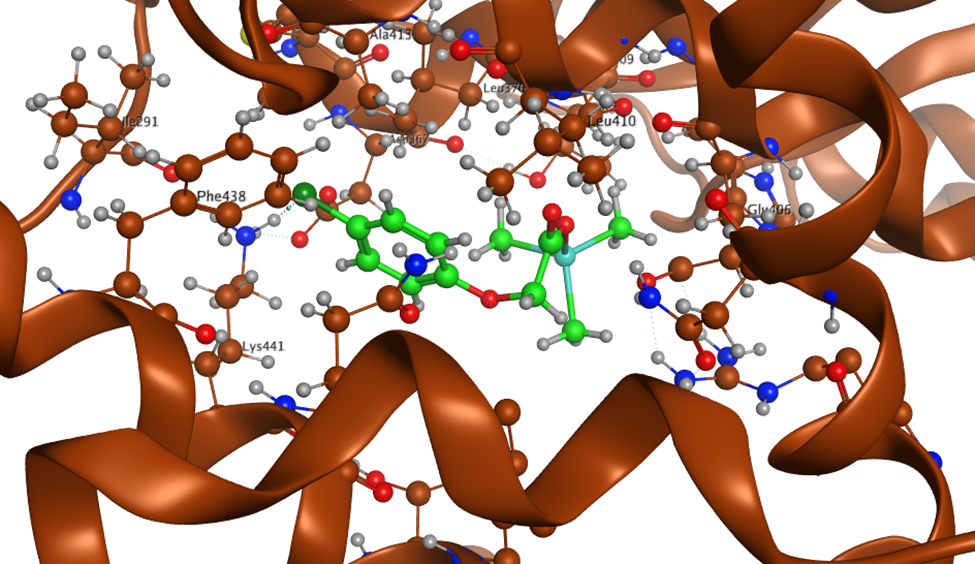


**Supplementary Figure 15.** Complex **4** interaction with angiotensin converting enzyme (ACE2) of human.


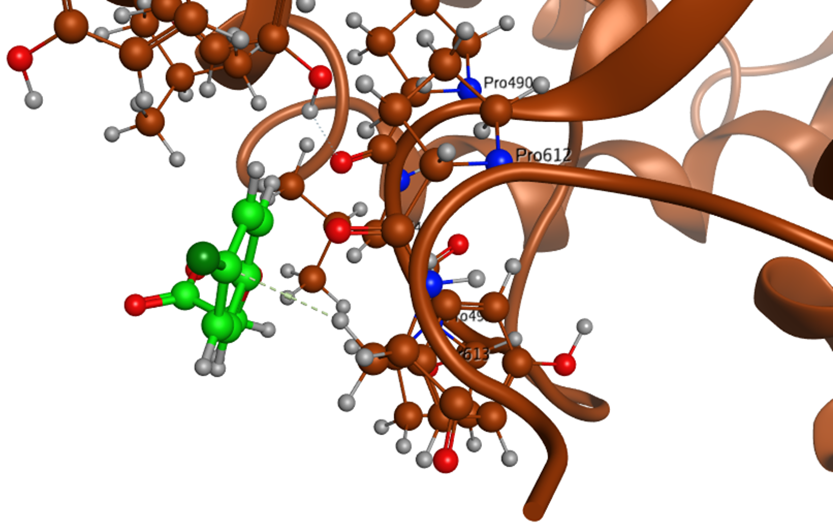


**Supplementary Figure 16.** Ligand acid (**HL**) interaction with angiotensin converting enzyme (ACE2) of human.

**Supplementary Table 1. Cell Viability (%) data of 1-4 against A549 and MRC-5 at different conc. (µg/mL)**

| **Viability (%)** | **A549** | | | | **MRC5** | | | |
| --- | --- | --- | --- | --- | --- | --- | --- | --- |
|  | **24h** | **SD** | **48h** | **SD** | **24h** | **SD** | **48h** | **SD** |
| **HL** | | | | | | | | |
| **0.0** | 100.00 | 6.31 | 100.00 | 3.39 | 100.00 | 8.15 | 100.00 | 6.63 |
| **0.1** | 96.01 | 3.63 | 94.85 | 4.67 | 87.74 | 1.81 | 94.90 | 4.30 |
| **0.5** | 103.38 | 8.65 | 99.11 | 7.04 | 98.16 | 3.72 | 115.17 | 3.73 |
| **1** | 83.84 | 27.87 | 100.69 | 2.36 | 108.00 | 1.82 | 113.25 | 2.28 |
| **2** | 90.23 | 20.33 | 104.34 | 2.15 | 103.81 | 4.27 | 118.56 | 4.45 |
| **5** | 69.23 | 9.18 | 98.45 | 2.01 | 101.65 | 9.17 | 114.07 | 7.96 |
| **10** | 97.82 | 15.31 | 97.19 | 2.42 | 101.26 | 7.97 | 117.63 | 7.08 |
| **50** | 81.54 | 2.26 | 83.30 | 2.40 | 104.60 | 5.29 | 105.95 | 9.86 |
| **Complex 1** | | | | | | | | |
| **0.0** | 100.00 | 6.31 | 100.00 | 3.39 | 100.00 | 8.15 | 100.00 | 6.63 |
| **0.1** | 95.88 | 11.87 | 93.95 | 3.90 | 82.46 | 7.14 | 97.73 | 7.73 |
| **0.5** | 92.91 | 15.92 | 90.21 | 8.64 | 98.62 | 4.87 | 111.22 | 4.90 |
| **1** | 89.13 | 25.32 | 99.47 | 4.91 | 95.97 | 6.17 | 104.10 | 7.00 |
| **2** | 51.79 | 3.05 | 95.17 | 1.28 | 92.81 | 4.40 | 109.18 | 4.83 |
| **5** | 75.81 | 18.10 | 96.49 | 2.36 | 99.93 | 4.55 | 101.50 | 8.11 |
| **10** | 96.64 | 2.57 | 102.96 | 3.10 | 94.86 | 5.74 | 105.95 | 6.22 |
| **50** | 71.76 | 0.90 | 87.19 | 3.92 | 99.34 | 3.54 | 93.74 | 16.48 |
| **Complex 2** | | | | | | | | |
| **0.0** | 100.00 | 6.31 | 100.00 | 3.39 | 100.00 | 8.15 | 100.00 | 6.63 |
| **0.1** | 89.96 | 15.34 | 96.40 | 3.21 | 60.52 | 3.08 | 65.30 | 3.07 |
| **0.5** | 103.29 | 10.50 | 98.78 | 2.96 | 59.89 | 3.32 | 65.15 | 2.64 |
| **1** | 96.12 | 17.59 | 94.71 | 1.02 | 65.34 | 4.17 | 63.19 | 2.65 |
| **2** | 102.32 | 7.47 | 85.55 | 2.58 | 92.20 | 3.99 | 86.98 | 6.82 |
| **5** | 50.67 | 12.06 | 46.55 | 2.47 | 100.80 | 7.00 | 98.42 | 4.74 |
| **10** | 31.69 | 1.79 | 39.77 | 2.08 | 92.26 | 3.96 | 96.80 | 7.24 |
| **50** | 25.58 | 0.49 | 40.45 | 1.64 | 90.14 | 4.16 | 109.68 | 20.09 |
| **Complex 3** | | | | | | | | |
| **0.0** | 100.00 | 6.31 | 100.00 | 3.39 | 100.00 | 8.15 | 100.00 | 6.63 |
| **0.1** | 77.76 | 8.88 | 86.64 | 2.65 | 62.55 | 1.88 | 61.69 | 2.52 |
| **0.5** | 61.05 | 3.27 | 57.09 | 2.10 | 63.51 | 1.68 | 63.76 | 3.29 |
| **1** | 54.76 | 2.87 | 44.52 | 1.92 | 64.36 | 1.70 | 61.61 | 2.14 |
| **2** | 25.67 | 1.03 | 39.45 | 1.15 | 65.43 | 1.87 | 61.04 | 5.80 |
| **5** | 24.86 | 0.51 | 41.20 | 1.04 | 69.43 | 1.66 | 86.66 | 7.84 |
| **10** | 24.41 | 0.59 | 38.68 | 1.87 | 94.97 | 4.24 | 57.22 | 6.97 |
| **50** | 24.94 | 1.16 | 40.85 | 1.21 | 99.25 | 4.05 | 95.37 | 8.34 |
| **Complex 4** | | | | | | | | |
| **0.0** | 100.00 | 6.31 | 100.00 | 3.39 | 100.00 | 8.15 | 100.00 | 6.63 |
| **0.1** | 76.56 | 12.99 | 99.51 | 4.29 | 84.64 | 4.03 | 77.59 | 4.17 |
| **0.5** | 101.02 | 6.12 | 99.63 | 3.74 | 104.20 | 4.46 | 101.46 | 6.64 |
| **1** | 91.91 | 2.63 | 99.36 | 3.47 | 103.11 | 4.26 | 100.62 | 2.67 |
| **2** | 56.84 | 15.04 | 97.61 | 6.26 | 101.98 | 3.39 | 103.04 | 7.49 |
| **5** | 70.21 | 23.34 | 59.44 | 2.58 | 102.04 | 7.21 | 99.48 | 4.56 |
| **10** | 46.38 | 4.36 | 58.69 | 4.13 | 101.63 | 3.81 | 106.42 | 3.96 |
| **50** | 43.45 | 6.41 | 55.18 | 5.11 | 102.24 | 4.68 | 100.51 | 3.62 |
