# Supplementary material for: Synthesis, Characterization, Biological Activity and Molecular Docking Studies of Novel Organotin(IV) Carboxylates
Source: Front Pharmacol. 2022 Apr 5;13:864336. doi: 10.3389/fphar.2022.864336 (PMC9017761; doi:10.3389/fphar.2022.864336)
Supplement: Supplementary file 2 [file DataSheet1.docx]

Supplementary Material

**Optimized coordinates of complex 4 at B3LYP/6-31G*+LANL2DZ level of theory**

Cl -2.48014800 6.11434400 -0.00023300

O -6.06067900 -2.24279500 0.00045300

O -3.82191300 -2.44527900 -0.00011800

O -3.55553700 0.29549100 0.00058100

C -4.92104600 -1.77841400 0.00026700

C -4.88013500 -0.23187900 0.00038700

H -5.42535900 0.11584500 -0.88687400

H -5.42559900 0.11571300 0.88755000

C -3.38167200 1.64492800 0.00037900

C -2.04956200 2.09688700 0.00028000

H -1.23414800 1.37733100 0.00033000

C -1.77727400 3.45994900 0.00009600

H -0.74830900 3.80409100 0.00000900

C -2.83057400 4.37798300 0.00001400

C -4.15098500 3.94622800 0.00012200

H -4.96241500 4.66710800 0.00006900

C -4.43067300 2.57560600 0.00030900

H -5.46401300 2.24981600 0.00042000

Sn -1.66636500 -2.17744100 -0.00020300

Cl 9.28170100 0.57336600 0.00037400

O 0.56093500 -2.00110700 -0.00054100

O 0.69956100 0.26804200 0.00006700

O 3.38403900 0.16490700 -0.00040400

C 1.17328700 -0.86841700 -0.00022200

C 2.69960800 -1.08532700 -0.00009700

H 2.97160900 -1.67187900 0.88758300

H 2.97172200 -1.67240200 -0.88739000

C 4.73439900 0.16851500 -0.00020200

C 5.34235800 1.43807500 -0.00049900

H 4.69987700 2.31279700 -0.00086200

C 6.72516800 1.56500600 -0.00032400

H 7.18674500 2.54715500 -0.00055400

C 7.52033700 0.41577800 0.00015100

C 6.94045100 -0.84744700 0.00044600

H 7.56656900 -1.73394900 0.00080800

C 5.54809700 -0.97557800 0.00026900

H 5.10987200 -1.96638500 0.00049000

C -1.65724400 -1.31194500 1.95867700

H -1.05657400 -1.94812800 2.61904600

H -2.67140900 -1.23898900 2.35895400

H -1.20032400 -0.32015800 1.93323300

C -1.65782000 -1.31107700 -1.95870400

H -2.67211400 -1.23781800 -2.35858600

H -1.05743700 -1.94698500 -2.61959800

H -1.20077200 -0.31935300 -1.93293700

C -1.45730700 -4.31108600 -0.00027100

H -0.39955000 -4.59134800 0.00014700

H -1.94176200 -4.74124500 -0.88332900

H -1.94249200 -4.74100200 0.88247400

**
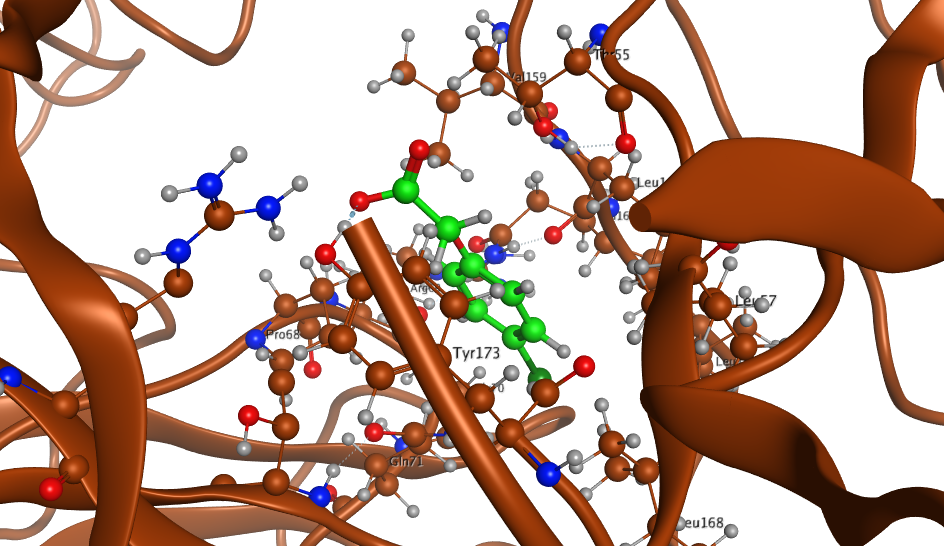
**

**Supplementary Figure 1.** Ligand acid (**HL**) interaction with nucleocapsid protein.

**
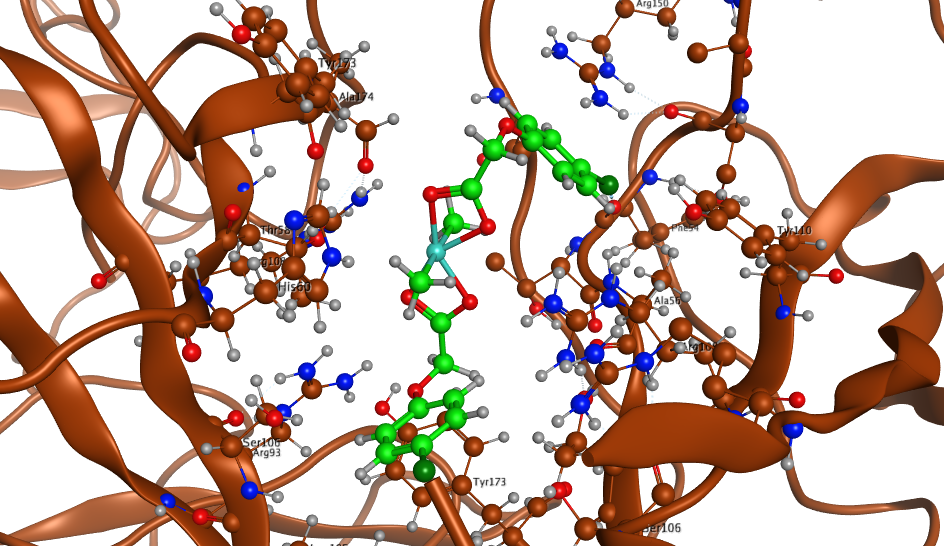
**

**Supplementary Figure 2.** Complex **2** interaction with nucleocapsid protein.

**
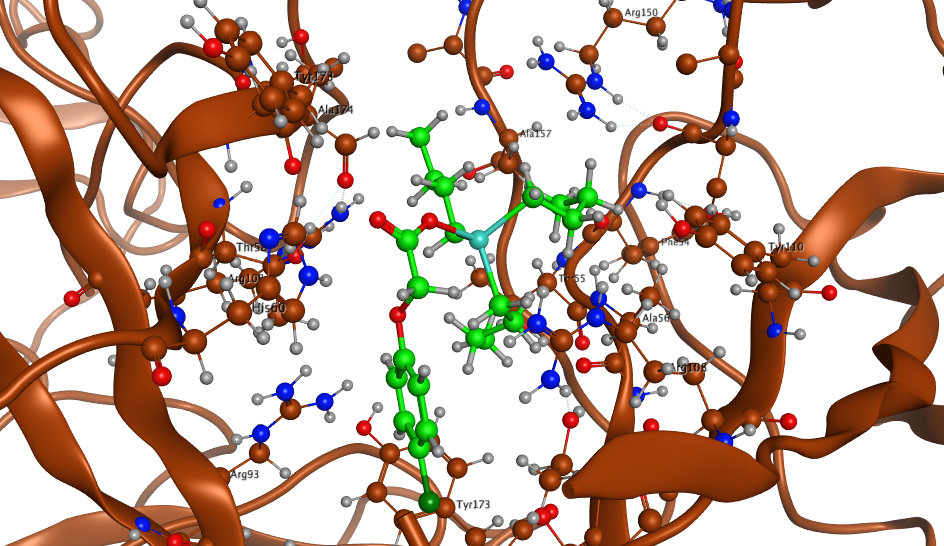
**

**Supplementary Figure 3.** Complex **3** interaction with nucleocapsid protein.

**
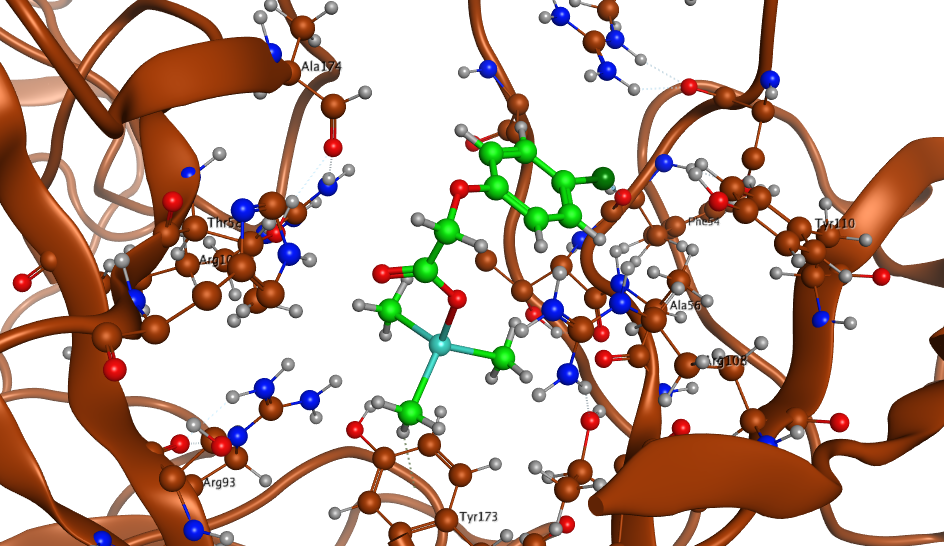
**

**Figure S4.** Complex **4** interaction with nucleocapsid protein.


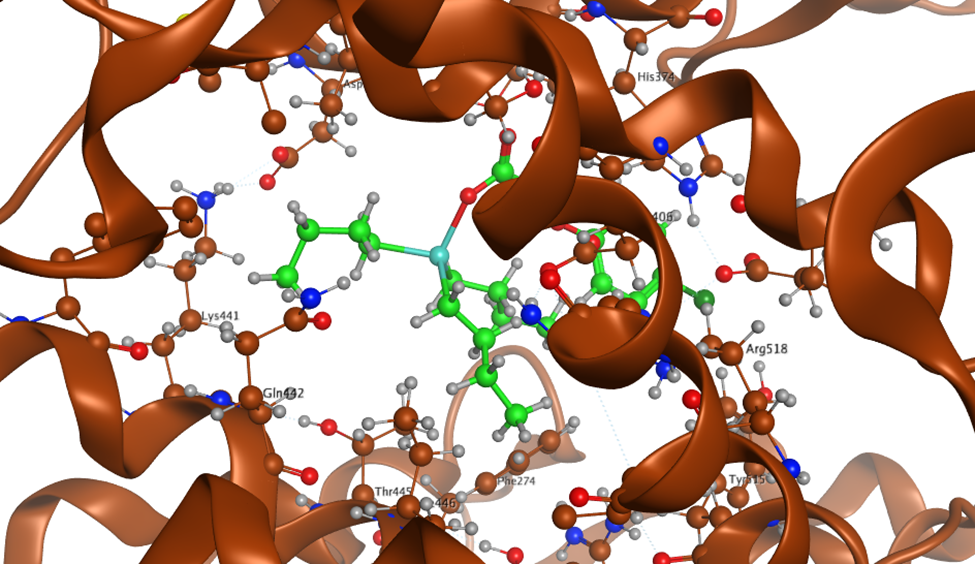


**Figure S5**.Complex **3** interaction with angiotensin converting enzyme (ACE2) of human.


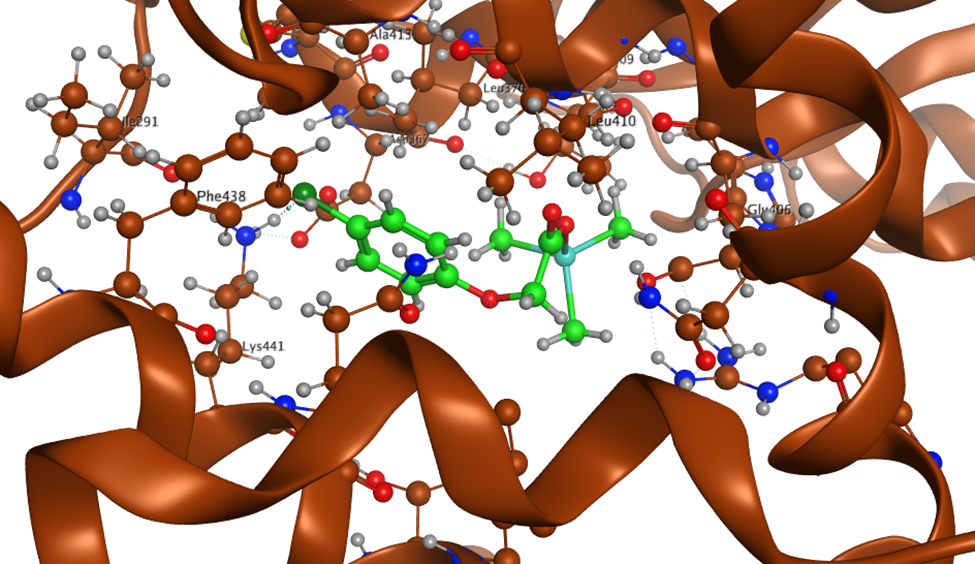


**Supplementary Figure 6**. Complex **4** interaction with angiotensin converting enzyme (ACE2) of human.


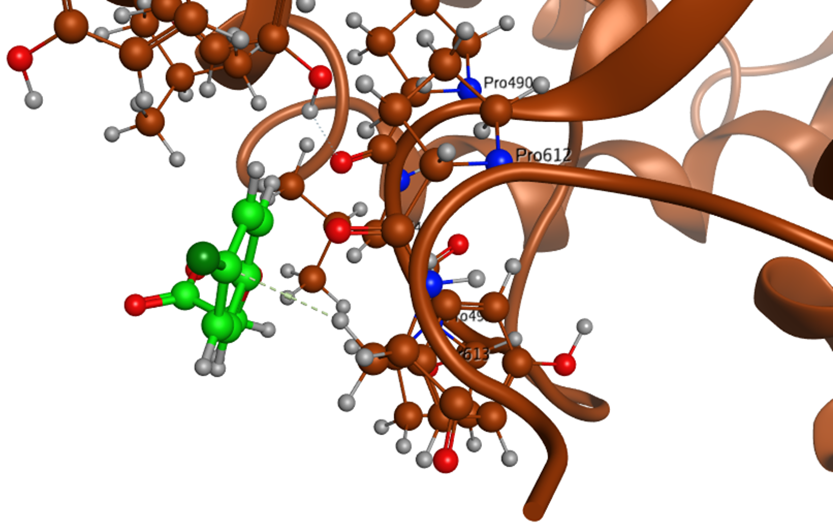


**Supplementary Figure 7**. Ligand acid (**HL**) interaction with angiotensin converting enzyme (ACE2) of human.
